# Early events in G-quadruplex folding captured by time-resolved small-angle X-ray scattering

**DOI:** 10.1093/nar/gkaf043

**Published:** 2025-01-30

**Authors:** Robert C Monsen, T Michael Sabo, Robert Gray, Jesse B Hopkins, Jonathan B Chaires

**Affiliations:** Department of Medicine, UofL Health Brown Cancer Center, University of Louisville, Louisville KY, 505 S Hancock St, Louisville, KY 40202, United States; Department of Medicine, UofL Health Brown Cancer Center, University of Louisville, Louisville KY, 505 S Hancock St, Louisville, KY 40202, United States; Department of Medicine, UofL Health Brown Cancer Center, University of Louisville, Louisville KY, 505 S Hancock St, Louisville, KY 40202, United States; The Biophysics Collaborative Access Team (BioCAT), Department of Physics, Illinois Institute of Technology, Chicago, IL 60616, United States; Department of Medicine, UofL Health Brown Cancer Center, University of Louisville, Louisville KY, 505 S Hancock St, Louisville, KY 40202, United States

## Abstract

Time-resolved small-angle X-ray experiments are reported here that capture and quantify a previously unknown rapid collapse of the unfolded oligonucleotide as an early step in the folding of hybrid 1 and hybrid 2 telomeric G-quadruplex structures. The rapid collapse, initiated by a pH jump, is characterized by an exponential decrease in the radius of gyration from 24.3 to 12.6 Å. The collapse is monophasic and is complete in <600 ms. Additional hand-mixing pH-jump kinetic studies show that slower kinetic steps follow the collapse. The folded and unfolded states at equilibrium were further characterized by SAXS studies and other biophysical tools, showing that G4 unfolding was complete at alkaline pH, but not in LiCl solution as is often claimed. The SAXS Ensemble Optimization Method analysis reveals models of the unfolded state as a dynamic ensemble of flexible oligonucleotide chains with a variety of transient hairpin structures. These results suggest a G4 folding pathway in which a rapid collapse, analogous to molten globule formation seen in proteins, is followed by a confined conformational search within the collapsed particle to form the native contacts ultimately found in the stable folded form.

## Graphical Abstract

**Graphical Abstract:**
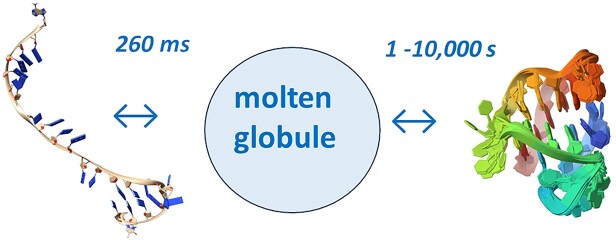

## Introduction

Understanding the folding mechanism of biological molecules is fundamental to understanding their form and function. G-quadruplexes (G4) are unique DNA or RNA structures whose folding, not surprisingly, differs in detail from the folding of proteins, RNA, or duplex DNA. G4 dynamics and the diversity of its folding mechanisms were recently reviewed [1–3]. The folding of G4 structures formed by human telomeric repeat sequences has been studied in the most detail by a variety of experimental approaches, including absorbance, fluorescence and circular dichroism spectroscopies, nuclear magnetic resonance (NMR), mass spectrometry, single-molecule methods, and molecular dynamics simulations [4–17]. No consensus on the detailed folding mechanism has emerged, apart from the fact that it is a multistep, slow process. Folding funnels, kinetics partitioning, and sequential folding models with populated intermediates were suggested by different investigators. Many specific questions about the process remain. Because of limitations in the time resolution of most of the methods used, early steps in the folding of telomeric sequences are poorly characterized. Stopped-flow absorbance studies inferred from the cation dependency of the fastest measurable relaxation time that a kinetic process faster than the 20 ms dead time of the instrument existed [6]. Stopped-flow absorbance, circular dichroism (CD), and fluorescence resonance energy transfer (FRET) studies from our laboratory [6, 11] captured early cation-driven folding events that were complete within 1–2 s. The FRET results indicated a fast reduction in the end-to-end distance of oligonucleotide chain, but no quantitative estimate of that distance was possible. A time-resolved CD spectrum was obtained by stopped-flow [11] of an intermediate species formed at 1 s with a spectrum characteristic of an antiparallel basket form [18] (or perhaps a two-tetrad basket antiparallel structure [19, 20]).

More recent microfluidic mixing [21, 22] and T-jump [23, 24] methods, with micro- to millisecond resolution, used FRET or CD detection to detect faster events. The mixing experiments were initiated by cation concentration jumps, and the fast events (20–40 μs lifetimes) observed were interpreted as the formation of hairpin loops. T-jump experiments monitored perturbations of the folded telomeric “basket” G4 structure in Na^+^ solution and monophasic fast relaxations in the ms to s range were observed over a range of cation concentrations and temperatures. The underlying molecular origin of these relaxations was not clear although it was noted that they were much slower than expected for simple hairpin formation. A new type of micromixer with a dead time of 40 μs and a wide time window allowing continuous data collection up to 300 ms detected multiple relaxation times by FRET for the folding the human telomere in KCl or NaCl [25]. The FRET signal was not converted to quantitative distance information. The interpretation of the multiple relaxation times as a sequential mechanism that includes a triplex intermediate is flawed by the failure of the authors to recognize that such a species forms only on a much slower time scale [11] than was measured using the new micromixer and that complete folding requires much longer than 300 ms [6, 11, 12, 14].

A full description of G4 folding remains beyond the reach of current molecular dynamics simulation methods. Unbiased MD simulation can at best reach a ms timescale, well below the actual experimentally observed timescale of the folding process. However, current MD results suggest that the folding process may be an extreme case of kinetic partitioning, and that the full G-quadruplex folding landscape is not reducible to a few order parameters or measurables. A number of prefolded structures, such as hairpins and triplexes, were calculated to be stable, and G-quadruplexes with strand-slippage were proposed as potential off-pathway intermediates [16]. Using NMR methods, Frelih *et al.* showed that, indeed, both strand-slipped triplex intermediates and 5′ hairpin features are observed to form from the telomere sequences under low temperature, low salt, and low pH conditions [26].

We report here the results of rapid-mixing, time-resolved small-angle X-ray scattering (TR-SAXS) experiments intended to better characterize the early events in G4 folding. The folding of both the human telomere hybrid 1, 5′TTGGG (TTAGGG)_3_A, (“2GKU”) and hybrid 2, 5′TAGGG (TTAGGG)_3_TT, (“2JSL”) structures in K^+^ solution was studied. A continuous laminar-flow TR-SAXS apparatus with millisecond resolution [27] was used to affect a pH jump to initiate G4 folding, with scattering profiles then collected at intervals over the range 1 ms to 1.2 s. The significant advantage of this TR-SAXS approach is that it allows rapid changes in a well-defined structural parameter, the radius of gyration (*R*_g_), to be monitored. To the best of our knowledge, this is the first use of time-resolved SAXS to characterize G4 folding.

## Materials and methods

### Oligonucleotide preparation

All oligonucleotides were purchased from Integrated DNA Technologies (IDT, Coralville, IA) as lyophilized, HPLC-purified and desalted powders. Stock solutions of 1 mM were prepared by dilution with MilliQ ultrapure water (18.2 MΩ × cm at 25°C) and stored at −20°C until use. Oligonucleotide concentrations were determined using the calculated molar extinction coefficients at 260 nm provided by IDT. All samples were further purified in their respective buffer by preparative size-exclusion chromatography (SEC) (Superdex 200 Increase 10/300 GL SEC column, GE Healthcare) prior to concentrating with Pierce protein concentrators (Thermo Fisher, #88515). Samples were annealed by diluting into their respective buffer to working concentration followed by heating for 20 min in 1 L of boiling water bath with slow cooling overnight to room temperature. Unless otherwise noted, all experiments were conducted in 8 mM potassium phosphate buffer supplemented with 185 mM KCl and pH adjusted to neutral (pH 7.2), alkaline (11.5), or acidic (pH 2.2) using either 1 M KOH or 1 M HCl.

### Circular dichroism

Normalized and background-corrected CD spectra were recorded in 1-cm quartz cuvettes at 20°C or 98°C with Jasco J710 or J810 spectropolarimeters adhering to the protocol outlined by Del Villar-Guerra [18]. Parameters were 220 to 320 or 340 nm wavelength range, 1.0 nm step size, 200 nm/min scan rate, 1.0 nm bandwidth, 2 s integration time, and four-scan accumulation. Spectra were base-line corrected for background and normalized by strand concentration using the following formula:


\begin{eqnarray*}\Delta \varepsilon = \theta /\ \left( {32982cl} \right)\end{eqnarray*}

where *θ* is ellipticity in millidegrees, *c* is molar DNA concentration in mol/l, and *l* is the path length of the cell in cm. For the pH-jump hand mixing experiments, 2GKU was annealed at 7.1 μM in pH 11.3 phosphate buffer and multiple “prejump” scans were measured to ensure it was denatured. At time 0 s, concentrated HCl was added by manual mixing with a pipette such that the pH was rapidly dropped to pH 7.1 (measured after the experiment) with a total dead time of ∼30 s.

### 1D nuclear magnetic resonance

1D ^1^H-NMR spectroscopy was performed on a Bruker Avance Neo 600-MHz instrument equipped with a nitrogen-cooled Prodigy TCI cryoprobe. 2GKU and 2JSL samples were prepared as above and concentrated to 0.5–1 mM in 250 μl volume using pre-rinsed Pall 3K MWCO concentrators prior to the addition of 5% (v/v) D_2_O. Measurements were made at 25°C in standard 3 mm NMR tubes. Minimization of water signal was achieved using a water flip-back pulse sequence.

For the pH-jump experiments, samples were prepared by annealing in alkaline phosphate buffer (pH 11.5) at ∼5 μM and concentrated to 2.2 mM in a volume of 500 μl using pre-rinsed Pall 3K MWCO concentrators, followed by addition of 5% (v/v) D_2_O. Experiments were performed at 25°C using a 5 mm NMR tube. Folding was initiated by adding 30 μl of a 1 M HCl solution to the NMR tube with gentle mixing using a stretched transfer pipette. The approximate dead time between mixing and measurement was 105 s. Minimization of water signal was achieved using a water flip-back pulse sequence. Hundred measurements were made over 693 s with four scans averaged per measurement. Measurements were repeatable by adjusting the pH back to 11.5 using the same %v/v addition of 1 M KOH and repeating the pH drop to neutral with additional 1 M HCl solution.

### Analytical ultracentrifugation

Analytical ultracentrifugation sedimentation velocity (AUC-SV) experiments were done using a Beckman Coulter ProteomeLab XL-A analytical ultracentrifuge (Beckman Coulter Inc., Brea, CA) at 20°C and using standard two sector cells. Each experiment was done at 40k rpm, and 150 scans were collected over an 8-h centrifugation period. Data were analyzed using the program SEDFIT [28] in the continuous c(s) model. Buffer density was determined on a Mettler/Paar Calculating Density Meter DMA 55A at 20°C, and buffer viscosity was measured on an Anton Paar Automated Microviscometer AMVn. For the calculation of frictional ratio and molecular weight, 0.55 ml/g was used for both single-stranded and G4 partial specific volume [29, 30].

### Equilibrium (EQ)-SAXS

Size-exclusion chromatography coupled SAXS (SEC-SAXS) was performed at the BioCAT beamline (18ID) at the Advanced Photon Source (APS) at Argonne National Lab. Prepared samples were centrifuged and subsequently loaded onto an equilibrated Superdex 200 Increase 10/300 GL column (Cytiva) maintained at a flow rate 0.7 ml/min (see Supplementary Table S1 below) using an AKTA Pure FPLC (GE Healthcare Life Sciences). After passing through the UV monitor, the eluate was directed through the SAXS flow cell, which consists of a 1 mm ID quartz capillary with 20 μm walls. A co-flowing buffer sheath was used to separate the sample and the capillary walls, helping to prevent radiation damage [31]. Scattering intensity was recorded with a Pilatus3 × 1M (Dectris) detector placed 3.628 m from the sample, giving access to a *q*-range of 0.0044 to 0.35 Å^−1^. A series of 0.5-s exposures were acquired continuously during elution, and the data were reduced using the software BioXTAS RAW version 2.1.1 [32]. Buffer blanks were created by averaging regions flanking the elution peak and subtracted from exposures selected from the sample elution peak to create the buffer corrected *I*(*q*) versus *q* curves for subsequent analyses. SAXS sample preparation, data collection, data reduction, analysis, presentation, and interpretation have been done in close accordance with recently published guidelines [33]. All SAXS data have been deposited in the SASBDB (https://www.sasbdb.org/) [34].

### TR-SAXS

Time-resolved SAXS (TR-SAXS) studies were performed at the BioCAT beamline (18ID) at the Advanced Photon Source (APS) at Argonne National Lab. DNA samples were prepared in pH 11.5 buffer as described above and concentrated to 9 mg/ml. The mixing buffer was a matched phosphate buffer at pH 2.2. TR-SAXS studies utilized a 5-inlet laminar flow mixing device [35] capable of measuring events on the 1–1500 ms timescale. The central channel contained the DNA samples, the diagonal channels contained the pH 11.5 sample buffer, and the vertical channels contained the pH 2.2 mixing buffer. Hand mixing experiments mimicking the conditions of the TR-SAXS experiment were conducted prior to analysis to ensure that the final pH upon mixing would be ∼7.2. Using 2GKU, a concentration series was conducted at 8.7, 4.0, and 2.0 mg/ml to evaluate ideal scattering conditions. An estimation of the mixing time of the laminar flow cell was calculated using equation (8) from [36] to ensure adequate mixing prior to the earliest time points. Mixing and pH equilibration were complete by collection of the first time point, obviating any need for signal deconvolution. It was determined that 4 mg/ml achieved the best *S/N* and agreement with equilibrium (EQ)-SAXS results. Data were collected over ∼1 ms to 1.2 s with 118 time points. For background subtraction, the mixing buffers were scanned without injection of the sample and used to subtract from the sample scans. Reported results are for 2GKU and 2JSL at 4.0 mg/ml.

Standard data reduction methods were used for radial averaging of each measurement. During some of the runs, the first few (time) points were missed due to slight misalignment of the sample stream with the beam path and so multiple runs at 4.0 mg/ml were conducted and averaged to obtain the final data sets. This is the reason that the *I*(0) plots show some sharp discontinuities, as *I*(0) is related to the concentration of sample. We also note that some data points in the low *q* range appear to sharply curve up, possibly indicating aggregation. However, orthogonal methods (NMR and AUC) show no detectable aggregation or oligomerization with the 2GKU and 2JSL sequences at these concentrations. The averaged data sets were analyzed using REGALS [37] in the BioXTAS RAW v2.2.2 [38] software with the EQ-SAXS data of the neutral and alkaline denatured sequences used to anchor at the beginning and end of the TR-SAXS data sets.

### Molecular dynamics and modeling

Starting coordinates of the single-stranded 2GKU and 2JSL sequences were generated in UCSF Chimera [39] using the “build” functionality. The PDB structures were then imported into the tleap module of AMBER 2020 to generate the sander input files. All simulations were done using the Generalized Born implicit solvation model (igb = 8) and equilibrated using sander at 300 K and 1 atm using the following steps: (i) minimization with weak restraints of 10.0 kcal/mol/Å on all nucleic acid residues (2000 cycles of minimization, 500 steepest decent before switching to conjugate gradient) and 16.0 Å cutoff, (ii) heating from 0 K to 100 K over 20 ps with 50 kcal/mol/Å restraints on all nucleic acid residues, (iii) minimization of the system without restraints (2500 cycles, 1000 steepest decent before switching to conjugate gradient) with 16 Å cutoff, (iv) heating from 100 K to 300 K over 20 ps with weak restraints of 10.0 kcal/mol/Å on nucleic acid residues, and (v) equilibration at 1 atm for 100 ps with weak restraints of 10.0 kcal/mol/Å on nucleic acids. The resulting coordinate files from equilibration were then used as input for duplicate 500 ns of unrestrained MD simulations using pmemd with GPU acceleration in the isothermal isobaric ensemble (*P* = 1 atm, *T* = 300 K) with the DNA OL15 force field. Random initial velocities were achieved using unique seeds based on the time of the simulation (ig = -1). Time steps (2.0 fs) were used with bonds involving hydrogen frozen using SHAKE (ntc = 2). Trajectories were analyzed using the CPPTRAJ module in the AmberTools20 [40] package. Model figures were made using UCSF Chimera v1.17.3 [39].

Molecular model ensembles were derived using the Ensemble Optimization Method 2.1 [41] program from the ATSAS suite of tools. A total of 10 000 snapshots equally spaced across the two 500 ns combined trajectories for both 2GKU and 2JSL were used to generate models that were included in each Ensemble Optimization Method (EOM) pool (i.e. a total of 10 000 models made up each respective pool). GAJOE was used in pool “-p” mode, with maximum curves per ensemble set to 20, minimum curves per ensemble set to 5, constant subtraction allowed, curve repetition allowed, and the genetic algorithm (GA) repeated 100 times. In brief, EOM takes a large pool of macromolecules covering as much conformational space as possible (and reasonable) and selects from this pool a sub-ensemble of conformers that recapitulate the experimental scattering. The ensemble is the subset of weighted theoretical curves from conformations that minimizes the discrepancy χ^2^:


\begin{eqnarray*}{{\chi }^2} = {\mathrm{\ }}\frac{1}{{K - 1}}{\mathrm{\ }}\mathop \sum \limits_{j = 1}^K {{\left[ {\frac{{\mu I\left( {{{s}_j}} \right) - {{I}_{{\rm exp}}}{\mathrm{\ }}\left( {{{s}_j}} \right)}}{{\sigma {\mathrm{\ }}\left( {{{s}_j}} \right)}}} \right]}^2}\end{eqnarray*}

where *I*_exp_ (*s_j_*) is the experimental scattering, *I* (*s_j_*) is the calculated scattering, *K* is the number of experimental points, *σ* (*s_j_*) are standard deviations, and *μ* is a scaling factor [42]. Ten independent EOM runs were performed for each system to ensure that convergence was achieved.

Calculations of 5′ and 3′ distances were performed in UCSF Chimera [39] using the “distance” command. Solvent accessible surface area (SASA) values were determined using the program FreeSASA [43] using default atom radii, slices [20], and a probe size of 1.400 Å.

## Results and analysis

### Equilibrium conformational analysis of the folded, unfolded, and Li^+^ destabilized states

To understand the telomere G4 folding pathway, we first identified conditions that favor the folded or fully unfolded forms. Unfortunately, chemical denaturants like urea and guanidine-HCl commonly used to unfold proteins or RNAs that do not unfold G4 structures [44]. G-quadruplexes are stabilized by a number of factors, including loop length, crowding effects, pH, temperature, and ionic strength and composition. G4 formation is mainly driven by the hydrogen bonding (Hoogsteen bonding of guanines in tetrads and loop “capping” structures that span either terminus), electrostatics (ion coordination in both the central G-tetrad channel and along the negatively charged backbone), and nucleotide stacking (pi-stacking of guanines and van Der Waals stabilization). The particular conformation of a G4 strongly depends on the identity of the central channel cation. For instance, the human telomere G4 sequence 143D adopts an antiparallel conformation in the presence of Na^+^ cations in solution [45], a parallel conformation in the presence of K^+^ cations in dehydrated, crowded crystallization conditions [46], and an equilibrium of hybrid 1 and hybrid 2 conformations in the presence of K^+^ cations in solution [47–49]. Other cations, such as Li^+^, have been reported as having destabilizing effects on G4 stability [50], with Li^+^ often used in place of K^+^ and Na^+^ ions in hopes of preventing G4 formation [26].

**Figure 1.**
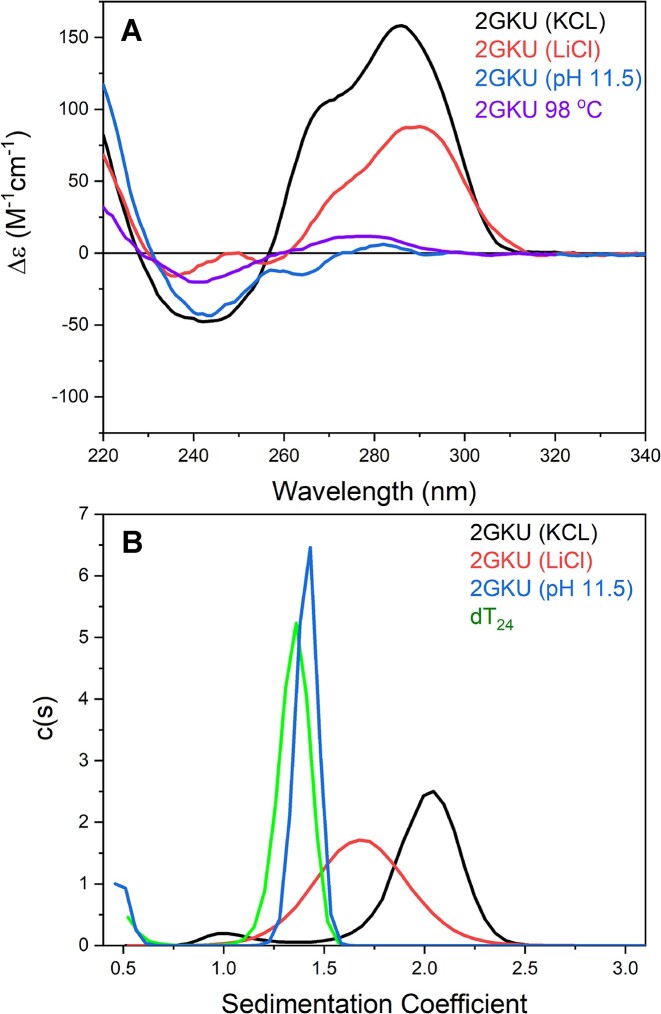
(**A**) CD spectra of 2GKU in KCL (black), LiCl (blue), KCl at pH 11.5 (purple), and KCl at 98°C (red). (**B**) Sedimentation coefficient distributions for 2GKU in KCl (black), LiCl (blue), and KCl at pH 11.5 (purple). The sedimentation of an unstructured single-stranded reference oligonucleotide, T_24_, is shown in green.

**Figure 2.**
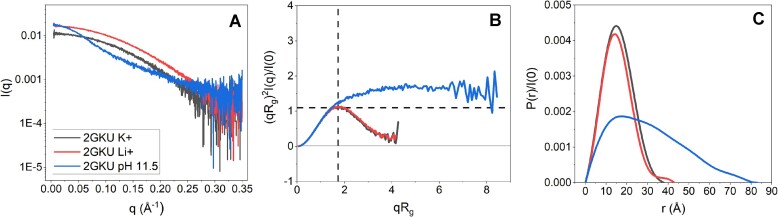
Equilibrium SAXS analysis of 2GKU. (**A**) Scattering profiles, (**B**) dimensionless (rebinned) Kratky plots, and (**C**) normalized *P*(*r*) distributions of 2GKU in KCl at pH 7.2 (black), LiCl at pH 7.2 (red), and KCl at pH 11.5 (blue).

Figures 1 and 2 and Supplementary Fig. S1 present the results of equilibrium studies characterizing the folded form of 2GKU in KCl solution compared to its alkaline and thermally denatured forms, to a reference single-strand oligonucleotide (dT_24_), and to 2GKU in LiCl solution. The CD spectra in Fig. 1A show the characteristic spectrum of the hybrid 1 folded form for 2GKU in KCl [18], in contrast to the nearly flat, featureless spectra for the alkaline and thermally denatured forms. Replacing K^+^ ions with Li^+^ (red curve) results in a spectrum with a near zero magnitude at 240 nm, but with significant signal in the range of 260–300 nm, indicating that nucleotide stacking interactions persist [51]. The observed spectrum is inconsistent with any reported G4 fold [18]. Supplementary Fig. S1 shows the matched ^1^H-NMR spectra for each condition. Only in KCl solution, 2GKU shows the characteristic 12 G-tetrad resonances at 10–12 ppm expected for a folded, hybrid 1 G4. Under alkaline denaturation conditions, no secondary structure proton shifts are observed in the ∼8–14.5 ppm range. In Li^+^ buffer at pH 7.2, 2GKU is not entirely denatured, as evident by the weak signals observed in the G-tetrad imino fingerprint region (10–12 ppm), some of which overlap with the chemical shifts of the folded hybrid 1 conformation. The same is observed for 2JSL under identical conditions (Supplementary Fig. S2).

Although heat denaturation may be effective for ensuring a fully unfolded G4 sequence, temperature jump kinetic experiments are difficult in practice, and temperature directly affects molecular diffusion, complicating folding analysis [52]. Also, orthogonal methods like SAXS, AUC-SV, and NMR are difficult at the high temperatures required to melt G4 DNA. Therefore, alkaline denaturation was chosen for subsequent folding analyses. The use of alkaline denaturation in kinetics studies of duplex DNA formation has a long history [53].

Figure 1B shows the results of AUC-SV analysis of the 2GKU sequence in K^+^ buffer at pH 7.2 and 11.5, Li^+^ buffer at pH 7.2, and an oligonucleotide of similar length to 2GKU consisting of only thymine, d(T)_24_, which was also in K^+^ buffer at pH 7.2. The fully folded hybrid 1 form of 2GKU (black line) sediments with *S*_20,w_ = 2.0, whereas at pH 11.5 its sedimentation is closer to the d (T)_24_ sequence (1.6 S_20,w_ versus 1.4 S_20,w_), indicating that it is unfolded. In the presence of Li^+^, 2GKU sediments at a rate intermediate to the fully unfolded and folded extremes (1.8 S_20,w_), indicating that it is more extended than the folded 2GKU, yet more compact than the pH denatured 2GKU, possibly existing as a fast equilibrium between the compact and extended states. The same trend is observed for 2JSL (Supplementary Fig. S3).

We next evaluated the global structural features of 2GKU in the same conditions using equilibrium SAXS. Figure 2A–C shows the background subtracted scattering, dimensionless Kratky, and *P*(*r*) distribution plots, respectively, for 2GKU at pH 7.2 with KCl (black), LiCl (red), and pH 11.5 with KCl (blue) (Supplementary Figs. S4–S9 show each SEC-SAXS elution profile). Kratky plots are mathematical transformations of the scattering data that yield semi-quantitative information about a particle’s flexibility or disorder. Globular nonflexible particles will have bell-shaped curves with a peak at ∼q*R*_g_ = √3 = 1.73 and peak height of 3/$e$ = 1.1, whereas flexible and disordered particles will exhibit elevated peak height and a characteristic plateau that extends out to higher values of qR_g_. The *P*(*r*) distribution is the real-space probability distribution of the inter-particle distances obtained from an inverse Fourier transform (IFT) of the scattering data, *I*(*q*). The *P*(*r*) distribution provides quantitative information, such as the radius of gyration and maximum particle dimensions, as well as three-dimensional structural information. For instance, a symmetrical bell-shape distribution occurs when the particle is globular, whereas a positive skew is indicative of elongated particles or particles with significant flexibility. An excellent practical guide to the interpretation of these plots can be found in [54].

Under alkaline conditions, 2GKU is maximally extended and flexible, given by the elevated plateau at high q*R*_g_ in its Kratky representation and right skew to more than double the *D*_max_ of the folded 2GKU in the *P*(*r*) plots. Denatured 2GKU exhibits nearly identical Kratky and *P*(*r*) distributions when compared to poly dT_24_ under neutral pH conditions (Supplementary Fig. S10). The measured *D*_max_ for alkaline denatured 2GKU is larger than the end-to-end distance of a rigid single-stranded sequence with the helicity found in a B-form conformation (experimental *D*_max,exp._ = ∼83 Å versus B-form calculated *D*_max,calc._ = 75 Å). Conversely, at pH 7.2 in the presence of either counterion, 2GKU is very compact and globular, given by the near symmetric bell and Gaussian shapes of the distributions, respectively. We have previously compared the K^+^-solution NMR structure of 2GKU to its equilibrium scattering and confirmed an excellent fit to the SAXS data [55]. An increase in *D*_max_ of 5 and 12 Å was observed for the LiCl condition for 2GKU and 2JSL (Table 1 and Supplementary Figs S2 and S11), respectively, in agreement with the increases in frictional coefficient (*f/f_o_*) observed in AUC-SV measurements.

**Table 1.** Physical properties of 2GKU and 2JSL under different solution conditions

Sequence	2GKU	2GKU	2GKU	2JSL	2JSL	2JSL
Property	KCl, pH 7.2	LiCl, pH 7.2	KCl, pH 11.5	KCl, pH 7.2	LiCl, pH 7.2	KCl, pH 11.5
*S*_20,w_	2.02	1.76	1.62	2.05	1.72	1.64
*f*/*f*_0_	1.37	1.52	1.79	1.41	1.51	1.81
*R*_s_ (Å)	16.9	18.3	22.5	17.8	28.2	23.2
*R*_g_, Å (Guinier)	12.27 ± 0.04	11.92 ± 0.01	24.26 ± 0.17	12.13 ± 0.03	12.89 ± 0.04	24.26 ± 0.11
*R*_g_, Å (GNOM)	12.23 ± 0.03	11.97 ± 0.02	24.56 ± 0.11	12.2 ± 0.03	13.18 ± 0.10	25.03 ± 0.08
*D*_max_, Å	38	43	83	39	51	87

**Figure 3.**
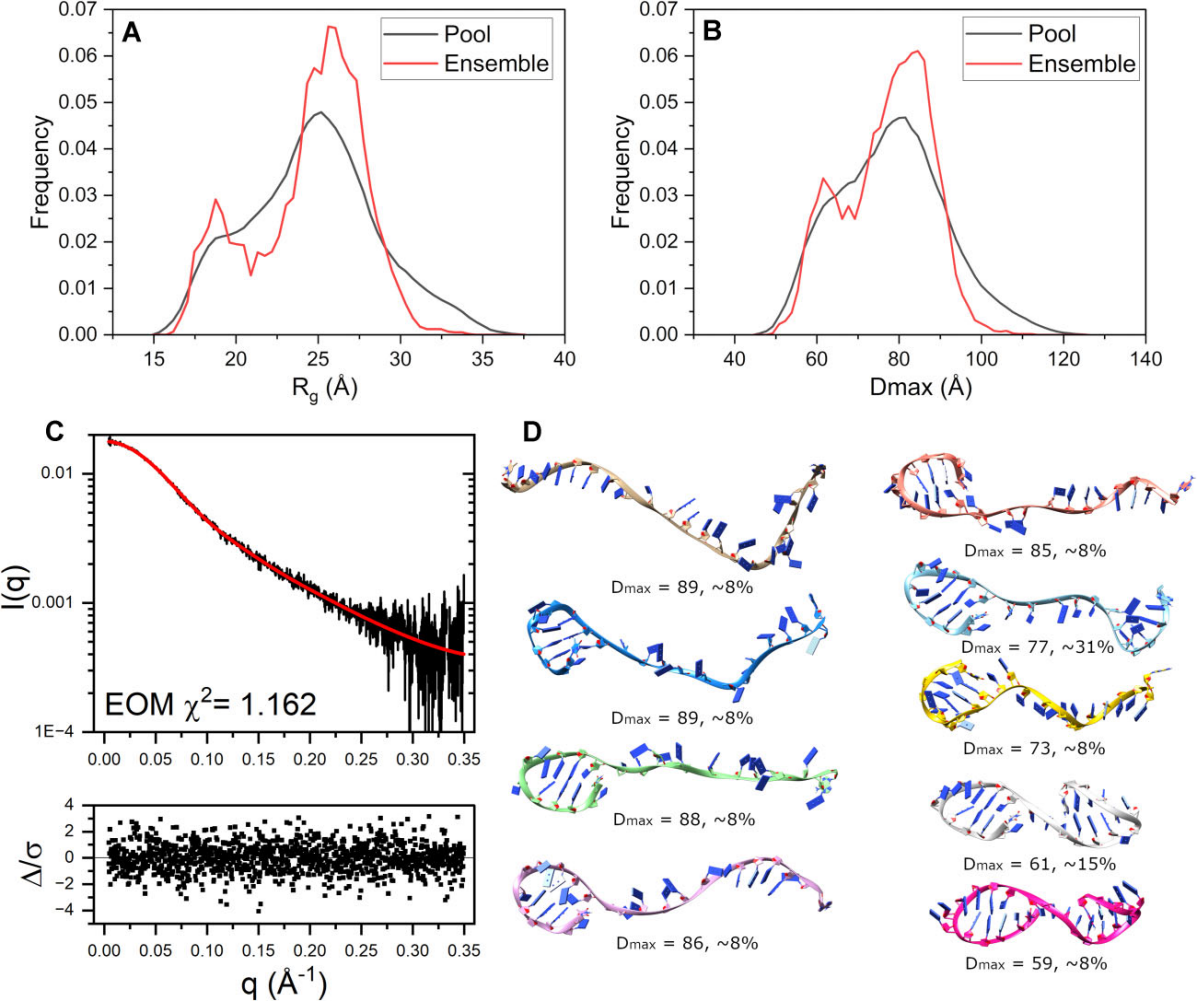
EOM analysis results for 2GKU. (**A** and **B**) EOM distributions for the radius of gyration (A) and *D*_max_ (B) for the total pool of conformers (black) and the selected ensemble of flexible structures (red). (**C**) pH 11.5 scattering curve with EOM fit overlaid in red and residuals below. (**D**) Best fit ensemble of conformers chosen by EOM from duplicate 500 ns implicitly solvated MD simulations starting from single-stranded 2GKU showing the most extended (top) to the most compact conformations (bottom) oriented with 5′ end on the left. Calculated *D*_max_ values and % ensemble weight are given below each model. EOM statistics: *R*_flex_ (random)/*R*_sigma_: ∼84.25% (∼90.67%)/0.85.

The exact nature of the denatured state is important for understanding the folding process, but little is known about it for G4 forming sequences. SAXS offers a unique capability for understanding its properties, as shown in Fig. 3. SAXS scattering curves are a weighted composite of all solution scatterers present. Therefore, flexibility as seen in Fig. 2 (blue curve) should be interpreted as an ensemble of flexible structures. To this end, we used the EOM [41], specifically the GAJOE GA, to determine a set of conformers from implicit solvent molecular dynamics simulations that have theoretical scattering curves that recapitulate the experimental data. In brief, EOM is an approach used to describe flexible or heterogeneous systems by selecting an ensemble of conformations from a pool of structures that recapitulate the experimental data. Unlike methods assuming a single static structure, EOM accounts for structural variability, providing insights into conformational distributions. This is achieved using a genetic algorithm, which iteratively selects, mutates and recombines ensembles of structural models from the pool to arrive at an optimized solution to the scattering curve. Figure 3A and B shows the EOM results as *R*_g_ and *D*_max_ distributions of the conformer pool (black lines) and selected ensemble (red lines), respectively. Figure 3C shows the scattering profile of the alkaline denatured 2GKU with an EOM ensemble fit overlaid (red) and with fit residuals shown below. The χ^2^-value of 1.162 and normally distributed residuals indicate a satisfactory fit of the ensemble of conformers shown in Fig. 3D. Moreover, the resulting representative ensembles have *R*_g_ values that are in excellent agreement with what was measured (2GKU: *R*_g,ensemble_= 24.56 Å versus *R*_g,experimental_= 24.56 ± 0.11 Å and 2JSL: *R*_g,ensemble_= 25.54 Å versus *R*_g,experimental_= 25.03 ± 0.08 Å). Representative 2GKU models in Fig. 3D are displayed from most to least extended going from top left to bottom right. The *D*_max_ range for the conformers is 59–89 Å and the fractional makeup is given in the figure for each model. Interestingly, most of the models constituting the EOM ensembles (∼92% weight) have guanine hairpin features that formed transiently throughout the simulations. 2JSL had a similar result, albeit with 100% of the ensemble having 5′ hairpin features included (Supplementary Fig. S12).

To further investigate the conformational distribution sampled in our MD studies, we extracted all inter-residue hydrogen bonds formed throughout the simulations and calculated their fractional occupancy across the simulation duration (Supplementary Figs. S13 and S14). The antiparallel 5′ guanine hairpins were more prevalent across both 2GKU and 2JSL simulations, consistent with prior MD studies [56] that show this as an early and semi-stable secondary feature. An antiparallel 3′ guanine hairpin also occurs but at a much lower frequency. No other secondary structures were observed over the duration of our simulations. We note that the distribution of radii of gyration sampled in both simulations span from ∼16 to 36 Å, indicating that the results from the EOM analysis were not biased by an under-sampled conformational space.

### TR-SAXS reveals millisecond timescale collapse to a prefolded intermediate

**Figure 4.**
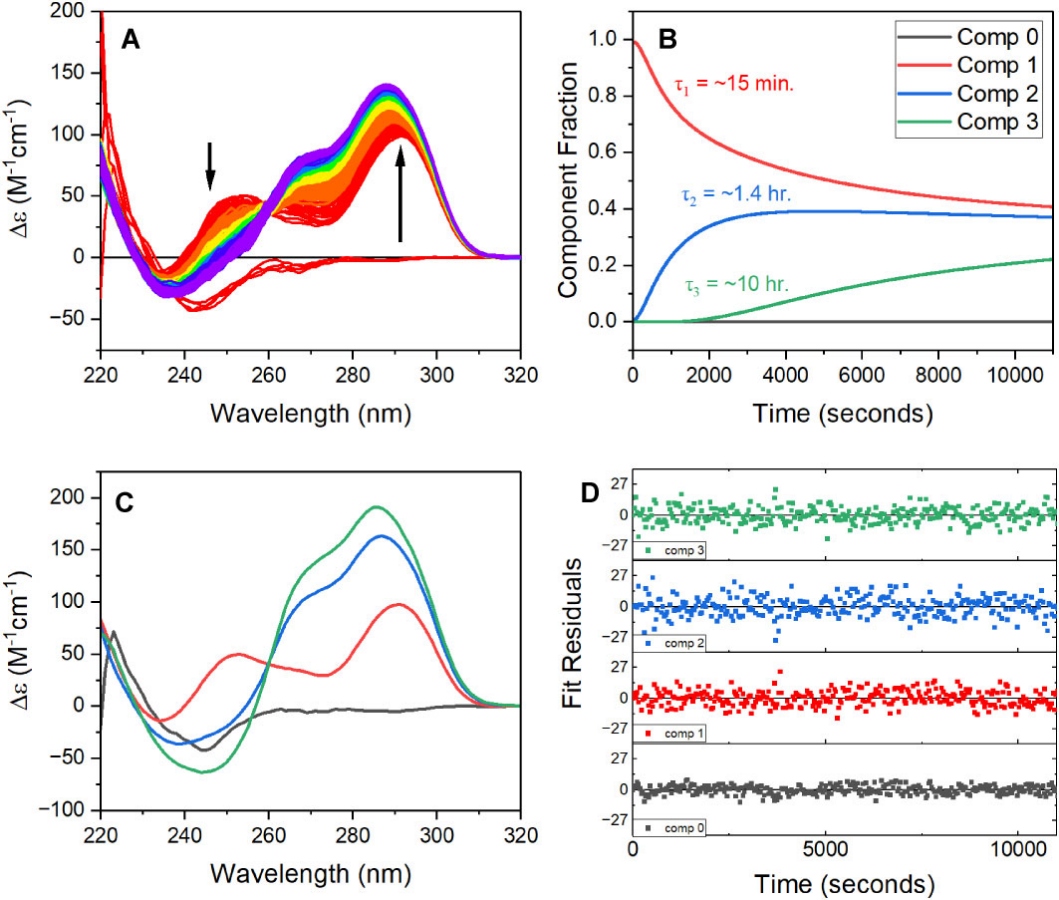
2GKU pH-jump spectra and kinetics. Folding was initiated by manually mixing a small volume of concentrated hydrochloric acid into the alkaline (pH 11.5) buffer solution to drop it to neutral pH (final pH 7.1). (**A**) CD spectra of the alkaline denatured 2GKU pre- and post-pH jump monitored over 11 000 s. (**B**) Fractional component plot from SVD with relaxation times overlaid based on single exponential fits. (**C**) Deconvoluted CD spectra for each component in B from SVD. (**D**) Residuals from the nonlinear least-squares fit to the data from SVD analysis.

Hand-mixing pH-jump experiments monitored by CD were done to ensure that a rapid adjustment from alkaline to a neutral pH gave folding kinetics that were consistent with previous cation-induced folding experiments [11]. Figure 4A shows the CD spectral changes of the 2GKU alkaline to neutral pH jump monitored over 11 000 s with a mixing dead time of ∼30 s. Analyzing the data using a singular value decomposition (SVD) and fits to a sequential folding model reveals that over this time frame two intermediates are formed with relaxation times of ∼875 ± 76 s and 5254 ± 328 s, rates in good agreement to those reported for cation-jump experiments [11]. The final spectrum is in quantitative agreement with what is expected for the folded hybrid 1 [18]. Upon the jump to neutral pH, component 1 (red) immediately forms and exhibits peaks at 250 and 290 nm that are consistent with spectral signatures of folding intermediates that have been previously observed by K^+^-induced folding of the same sequence [11] and a mass spectrometry kinetic study [14] that proposed a two-tetrad antiparallel basket conformation [20] as an intermediate. The previous kinetic studies investigating the K^+^-induced 2GKU folding at the millisecond timescale show that the earliest spectral intermediate forms within about 300 ms and exhibits a positive 290 nm peak and negative ellipticity at 260 nm [11], consistent with an antiparallel fold [57]. However, similar hand mixing pH-jump NMR experiments, deconvoluted with SVD, show complex imino shifts that are not easily assigned to discrete G4 folds (Supplementary Fig. S15). Overall, the pH-jump kinetics shown in Fig. 4 are fully consistent with the kinetics of potassium-jump experiments reported previously [11].

**Figure 5.**
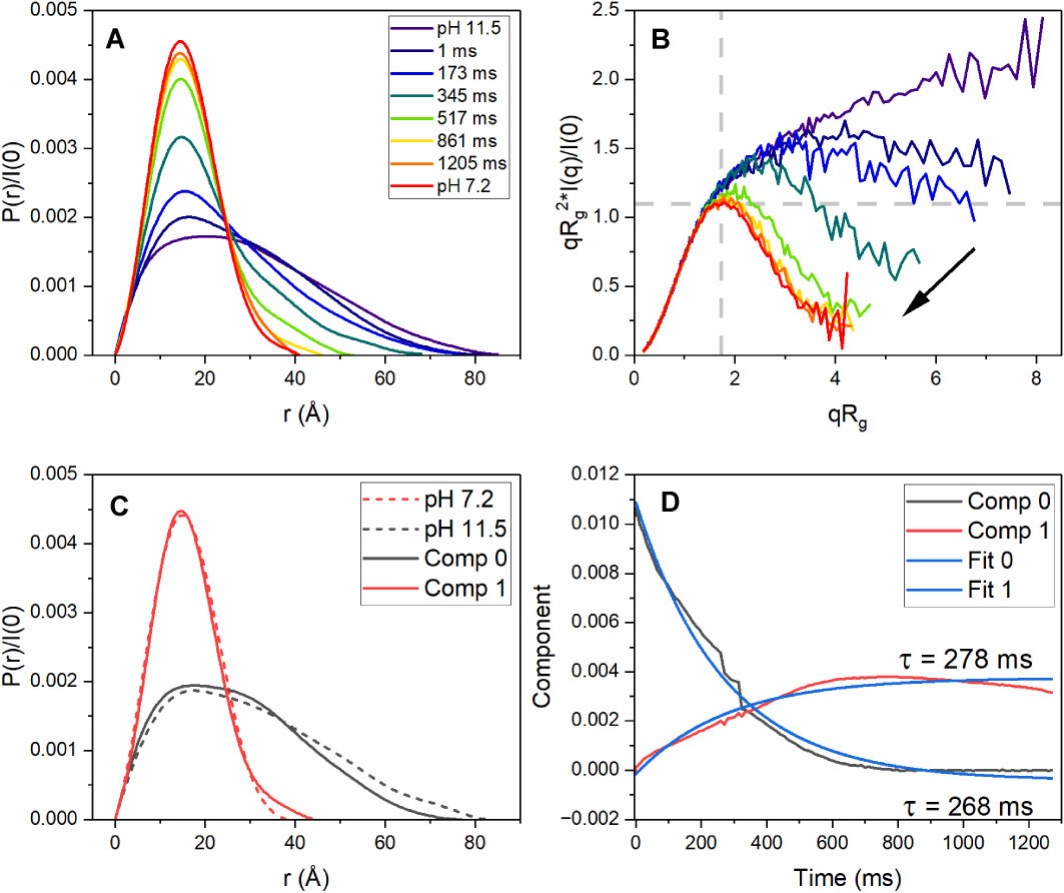
Time-resolved SAXS results of 2GKU following pH jump from 11.5 to 7.2. (**A**) Selected normalized *P*(*r*) distributions of the pH-induced structural collapse of 2GKU bracketed by the equilibrium SAXS profiles showing the conversion from an extended unstructured species to a globular and compact particle of nearly identical size and shape as the folded hybrid 1 form. (**B**) Dimensionless Kratky plots (data rebinned for clarity with log mode and rebin factor 4) showing the transition from the denatured flexible chain to a compact globular form that is nearly identical to the equilibrium 2GKU scattering. (**C**) REGALS derived regularized *P*(*r*) distributions comparing the two deconvoluted components (solid lines) with the equilibrium SAXS 2GKU distributions (dashed lines). (**D**) Regularized component concentration profiles from REGALS deconvolution with single exponential decay relaxation times overlaid and fits shown in blue. Red and black curves correspond to the solid red and black *P*(*r*) distributions in C.

Time-resolved SAXS experiments were conducted to provide insight into the structural collapse following imino protonation on a sub-second time scale. Figure 5A and B show the normalized *P*(*r*) distribution and dimensionless Kratky plots derived from the scattering time series for the pH-induced folding of 2GKU monitored over 1–1200 ms. For clarity, the Kratky distributions have been rebinned, and only select time points shown. From these plots it is evident that between time 0 s (i.e. the pH 11.5 equilibrium SAXS data) and ∼1 ms there is a noticeable reduction in *D*_max_ values commensurate with changes in the Kratky region of 3.6–8 q*R*_g_, suggesting that a sub-millisecond event occurs prior to the start of our TR-SAXS measurements. DNA hairpin formation occurs on timescales of microseconds or less [58], and recent TeZla micromixer fluorescence experiments of the FRET-labeled 143D telomere G4 (using K^+^-induced folding) have shown that the telomere hairpins appear in under 100 μs [25]. Our measurements may have missed these rapid hairpin formation events that occur within the dead time of mixing, although our equilibrium SAXS modeling with EOM clearly suggested their presence. Inspection of curves from the final time points (861 and 1205 ms) indicates that the collapse from the hairpin ensemble to globular prefolded intermediate is complete well within 1 s. Direct fitting of the derived *R*_g_ values versus time using a single exponential decay model reveals a relaxation rate of τ = 187 ± 13 ms (Supplementary Fig. S16) that is in fair agreement with values obtained by prior K^+^-induced folding measured by stopped-flow absorbance (τ_285 nm_ = 104 ± 4 ms, τ_265 nm_ = 260 ± 20 ms) [11].

As the TR-SAXS kinetic data set is complex and could contain unanticipated evolving species, we employed REGularized Alternating Least Squares (REGALS) [37] analysis within the BioXTAS RAW [32] package to deconvolve the time-resolved scattering data. An excellent explanation of REGALS and its use in deconvoluting complex SAXS profiles can be found in reference [37], and its implementation is also described on the BioXTAS RAW tutorial page (https://bioxtas-raw.readthedocs.io/en/latest/tutorial/s2_regals.html) [38]. In brief, REGALS is a method used to decompose SAXS data while incorporating constraints like non-negativity and smoothness to retrieve distinct structural features. Unlike standard SVD, which may produce physically meaningless components, REGALS ensures solutions are interpretable and aligned with known biophysical properties. In both 2GKU and 2JSL data sets only two components were identified from SVD, corresponding to the pre- and post-collapsed folding intermediates. REGALS was then run with our *D*_max_ values from equilibrium SAXS as estimated constraints to obtain the deconvolved spectra shown in Fig. 5C with corresponding component concentration profiles in Fig. 5D. The resulting *P*(*r*) distributions are physically reasonable and in good agreement with the equilibrium *P*(*r*) distributions (dashed lines). Fitting the component concentration curves in Fig. 5D with single exponential functions yields relaxation times of 278 and 268 ms for the formation of the collapsed structure and the disappearance of the prefolded structure, respectively. 2JSL also had only two components and the REGALS deconvolution showed a collapse with similar relaxation times of ∼290 ms (Supplementary Fig. S17). Again, these rates of collapse are in excellent agreement with previous spectroscopic stopped flow investigations [11] and the recent TeZla mixing device results using the FRET-labeled 143D [25].

The power of time-resolved studies by SAXS (and analysis with REGALS) is that along with the kinetic analyses and qualitative descriptions, there are also quantitative structural properties that aid in describing the nature of the pre- and post-collapse species. Table 2 compares the equilibrium and time-resolved SAXS *R*_g_ and *D*_max_ values for 2GKU and 2JSL. As discussed in the description of the *P*(*r*) and Kratky data above, the *D*_max_ and *R*_g_ values of the equilibrium alkaline denatured species are much larger than the earliest time points of the TR-SAXS measurements, which suggests sub-millisecond events are occurring, such as a shift to all hairpin structures. Similarly, although the collapse has finished by our latest time points, the *D*_max_ and *R*_g_ values of the TR-SAXS species are larger than for the fully folded and equilibrated 2GKU and 2JSL, indicating that their folding is not complete.

**Table 2.** Comparison of equilibrium (EQ) and time-resolved (TR-SAXS) results of 2GKU and 2JSL comparing the equilibrium denatured and folded results in KCl to the initial and final species from time-resolved SAXS. See also Supplementary Table S1

Sequence	2GKU				2JSL			
Property	EQ-SAXS (pH 7.2)	TR-SAXS (final)	EQ-SAXS (pH 11.5)	TR-SAXS (initial)	EQ-SAXS (pH 7.2)	TR-SAXS (final)	EQ-SAXS (pH 11.5)	TR-SAXS (initial)
*R*_g_, Å (GUINIER)	12.27 ± 0.04	12.63 ± 0.01	24.26 ± 0.17	20.65 ± 0.03	12.13 ± 0.03	12.80 ± 0.01	24.26 ± 0.11	21.57 ± 0.05
*R*_g_, Å (GNOM)	12.23 ± 0.03	12.84 ± 0.02	24.56 ± 0.11	21.63 ± 0.04	12.20 ± 0.03	13.02 ± 0.03	25.03 ± 0.08	22.40 ± 0.05
*D*_max_, Å	38	48	83	73	39	48	87	79

## Discussion

These data show that an early step in G4 folding, complete in <1 s, is the collapse from an extended, flexible, polynucleotide chain to a compact form. For the 2GKU sequence, the radius of gyration decreases from 24.3 to 12.6 Å, while the maximal interparticle distance decreases from 83 to 48 Å. The collapsed particle is slightly larger than the fully folded form at equilibrium (Table 2). These quantitative measures provide structural details that must be accounted for in any kinetic or computational model of the folding process. Kinetic steps both precede and follow this collapse. Events faster than the 1 ms dead time of the TR-SAXS apparatus may plausibly be attributed to transient hairpin formation, especially since the scattering curve for the unfolded state at equilibrium is best modeled as an ensemble of largely single-stranded forms with a variety of transient hairpins (Fig. 3). Kinetic studies with μs time resolutions have proposed hairpin formation as an initial step in G4 folding [21, 22, 25]. Following the rapid collapse, additional kinetic steps occur over 1–10 000 s that can be observed by UV absorbance, CD, FRET, NMR, and mass spectrometry [6, 9, 11, 12, 14]. These studies show that long-lived intermediates exist, since the observation of characteristic spectral or charge states requires that species accumulate at sufficient concentrations to produce a measurable signal. Specific cation coordination to stabilize the final folded G4 conformation seems to occur after the collapse reported here [14].

The conformational freedom of the unfolded polynucleotide chain provides a favorable entropy contribution that opposes G4 folding and stabilizes the denatured state. With rotation around six backbone torsion angles for each nucleotide, along with rotation around the glycosidic bond that attaches the base, an estimate for the Boltzmann conformational entropy of the 2GKU sequence (with *n* = 24 nt) is *S* = *Rln* (7^*n* − 1^) = *Rln* (7^23^) = 88.6 cal/K-mol, where *R* is the gas constant, and the exponential term enumerates the number of possible microstates. Experimental calorimetric measurements [59] of 2GKU unfolding determined a favorable entropy of 60.4 cal/K-mol for the first step in a sequential folding process, in good agreement with the theoretical estimate given that there are surely other coupled contributions within the experimental value. These entropy estimates suggest that the loss of conformational freedom presents a free energy barrier (Δ*G*_conf_ = −*T*Δ*S*) of 18–26 kcal/mol that must be overcome for folding. The favorable free energy of folding arises from favorable contributions from molecular interactions such as hydrogen bonding, base stacking, and cation coordination. But an often overlooked contribution from the hydrophobic force resulting from the removal of solvent accessible surface area also drives folding. The cited experimental calorimetric study [59] accurately determined the heat capacity change for the first step in the 2GKU folding process to be −420 cal/K-mol. Spolar and Record [60] determined an empirical relationship between the hydrophobic free energy contribution (Δ*G*_hyd_) and the heat capacity change (Δ*C*_p_), Δ*G*_hyd_ = 80 Δ*C*_p._ From this relation, a free energy contribution of −33.6 kcal/mol from the hydrophobic force overcomes the entropic barrier and drives the first step in the folding of 2GKU. The origin of this contribution is the removal of the SASA with a concomitant release of water. Hadzi and Lah [61] determined the correlation Δ*C*_p_= 0.134 (ΔSASA), where ΔSASA is the change in SASA, from which an estimate of the removal of ∼3100 Å^2^ of surface area results for 2GKU folding. Using the model weights from the EOM ensemble to more accurately model the SASA of the unfolded state (Fig. 3), we can approximate the weighted change in SASA for 2GKU’s collapse to be 2477 Å^2^. This estimate results in a predicted heat capacity change of Δ*C*_p_= 0.134 (−2477 Å^2^) = −332 cal/K-mol, which is close to the experimental measured value (i.e. −420 cal/K-mol), and Δ*G*_hyd_ = −26.6 kcal/mol, which is approximately the maximum estimated energy needed to overcome the entropic cost of folding. Our TR-SAXS measurements have, for the first time, provided the structural details and basis of this initial hydrophobic collapse.

Rather than offering a formal kinetic mechanism for the overall folding process, it is perhaps more useful to provide a phenomenological description of events that occur that must be accounted for in any mechanistic proposal. Below one millisecond, transient hairpin formation likely occurs that have been experimentally observed [62,63] and captured by our EOM model (Fig. 3). MD simulations seem to capture many of the bewildering ensemble of possible hairpins that might form in this phase [16]. A stable hairpin structure was detected by NMR in the 5′ end of the human telomere G4 sequence [26]. At ∼1 ms, the hydrophobic collapse described here to a “molten globule”-type structure occurs. The collapse is complete by 1 s. FRET changes occur on this same timescale indicating that the polynucleotide end-to-end distance is drastically decreased [11]. SAXS provides a quantitative description of the global structural features of this collapsed structure that could be used to test molecular simulations of the process (Table 2). Next, a time-resolved CD spectrum shows that by 2 s an intermediate has formed with the spectral characteristics of an antiparallel G4 structure [11]. Over the 5–1000 s timescale, FRET and CD changes are stable [11], and mass spectrometry (indicative of cation binding) shows the transient formation of a + 1 charge species that transforms into a + 2 charge species [14]. By 1000 s, the +1 charge species has disappeared, leaving only the +2 species. During this phase, there are complex, position-dependent, changes in the fluorescence of the site-specific probe 2-aminopurine substituted at specific locations in the G4 sequence, indicative of strand rearrangement [11]. The development of NMR spectra attributable to G4 hybrid structures begins ∼100 s (Supplementary Fig. S15), perhaps earlier but inaccessible because of the dead time of NMR mixing experiments used [12]. From 1000–10 000 s, NMR spectra evolve to a spectrum characteristic of a G4 hybrid 1 spectrum (like 2GKU). During this phase, subtle CD and 2-aminopurine fluorescence changes occur until equilibrium is reached [11]. Overall, during these phases discrete populated intermediates come and go, leading to the formation of G4 hybrid structures at the final equilibrium. Discrete intermediates must form in sufficient concentrations to provide the biophysical signals observed over these phases.

## Conclusion

**Figure 6.**
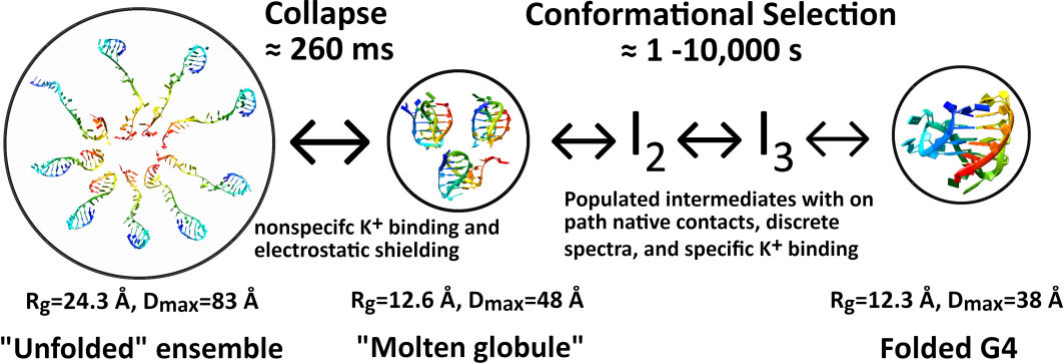
Telomere G4 folding is both a “funnel” and classical sequential kinetic mechanism. The figure shows a general overview of the collective steps in telomere G4 folding. The molecular models, aside from the folded G4 (2GKU, hybrid 1), are representative of the proposed ensembles described in the text. The black spheres surrounding each of the models serves to visually emphasize the collapse in radii of gyration at each measured step.

As an overall global description of the folding process (Fig. 6), we propose that the unfolded polynucleotide chain is in fact never fully extended but instead is an ensemble of transient hairpin structures that collapses into a “molten globule” structure at the onset of folding, driven primarily by hydrophobic forces. “Kinetic partitioning” may well describe this collapse. Within the collapsed particle, a conformational selection process may then occur within the confined space of the globule, with specific cation binding and coordination serving to stabilize on-pathway intermediates and the final native conformation. In this view, G4 folding is neither a kinetic partitioning phenomenon nor discrete sequential folding pathway—it is both.

## Supplementary Material

gkaf043_Supplemental_File

## Data Availability

Most data underlying this article are available in the article and in its online supplementary material. The time-resolved SAXS data underlying this article will be shared on reasonable request to the corresponding author.
