# Supplementary material for: Early events in G-quadruplex folding captured by time-resolved small-angle X-ray scattering
Source: Nucleic Acids Res. 2025 Jan 30;53(3):gkaf043. doi: 10.1093/nar/gkaf043 (PMC11780883; doi:10.1093/nar/gkaf043)
Supplement: gkaf043_Supplemental_File [file gkaf043_supplemental_file.docx]

**Supplemental Material: Early Events in G-quadruplex Folding Captured by Time-Resolved Small-Angle X-Ray Scattering.**

Robert C. Monsen^1,*^, T. Michael Sabo^1^, Robert Gray^1^, Jesse B. Hopkins^2^ and Jonathan B. Chaires^1,*^

1 Department of Medicine

UofL Health Brown Cancer Center, University of Louisville, Louisville KY

505 S Hancock St, Louisville, KY 40202

E-mail: rcmons01@louisville.edu, j.chaires@louisville.edu

2 The Biophysics Collaborative Access Team (BioCAT) Department of Physics

Illinois Institute of Technology, Chicago, IL 60616

*To whom correspondence should be addressed. Tel: +1 502 852 3653; Email: Robert.monsen@louisville.edu

Correspondence may also be addressed to Jonathan B. Chaires; Email: j.chaires@louisville.edu

**
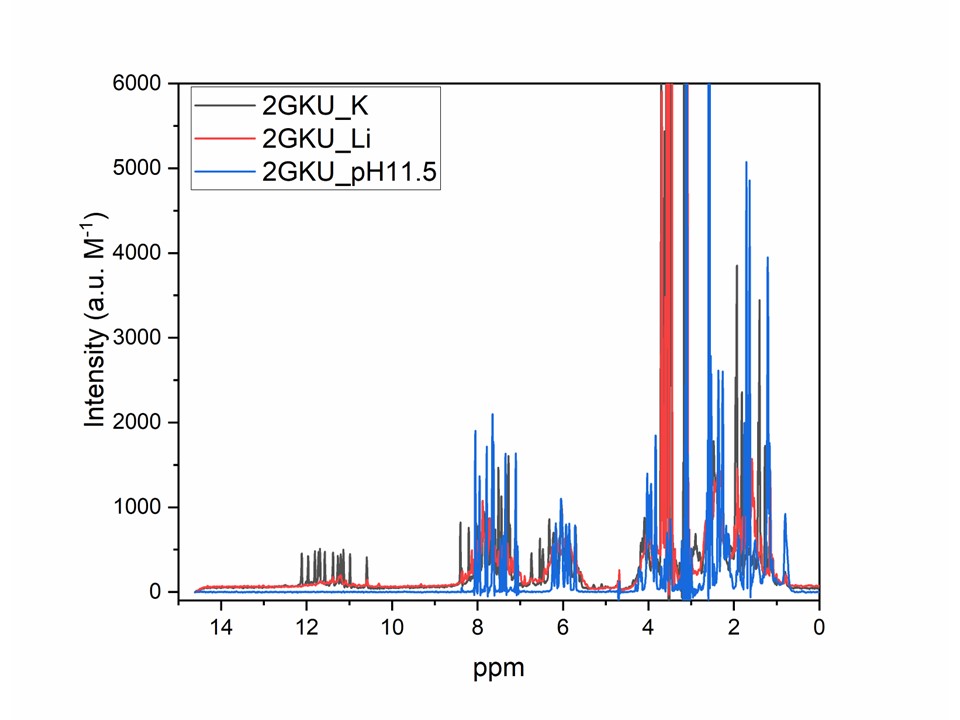
**

**Figure S1.** NMR spectra of 2GKU under various solution conditions. The alkaline denatured and Li^+^ conditions show a sharp reduction in the G4 imino shifts in the range of 10-12 ppm.


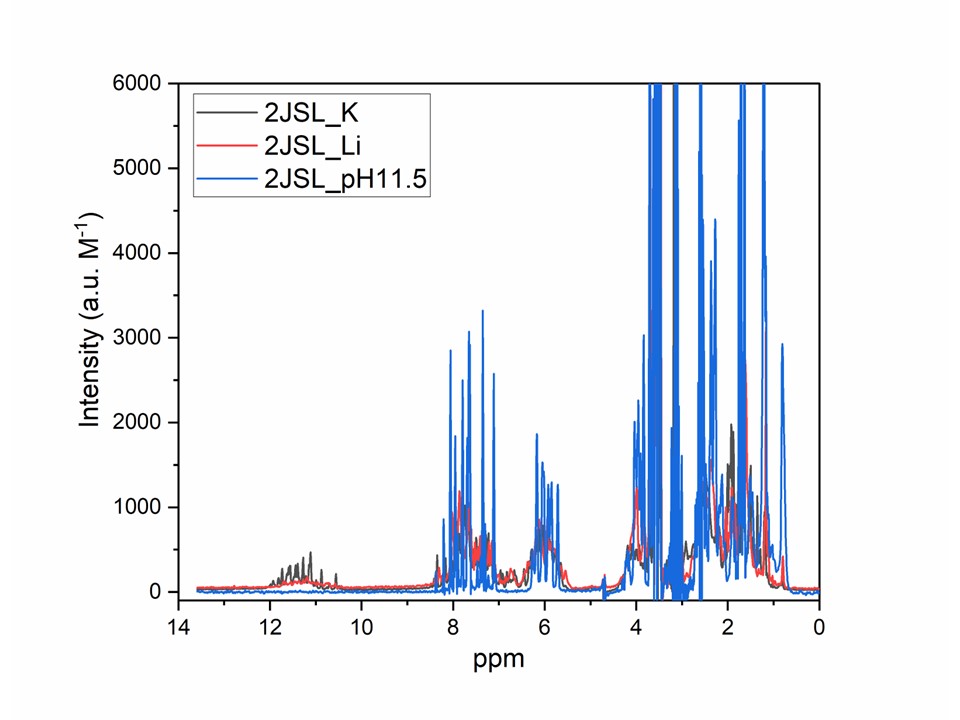


**Figure S2.** NMR spectra of 2JSL under various solution conditions. The alkaline denatured and Li^+^ conditions show a sharp reduction in the G4 imino shifts in the range of 10-12 ppm.


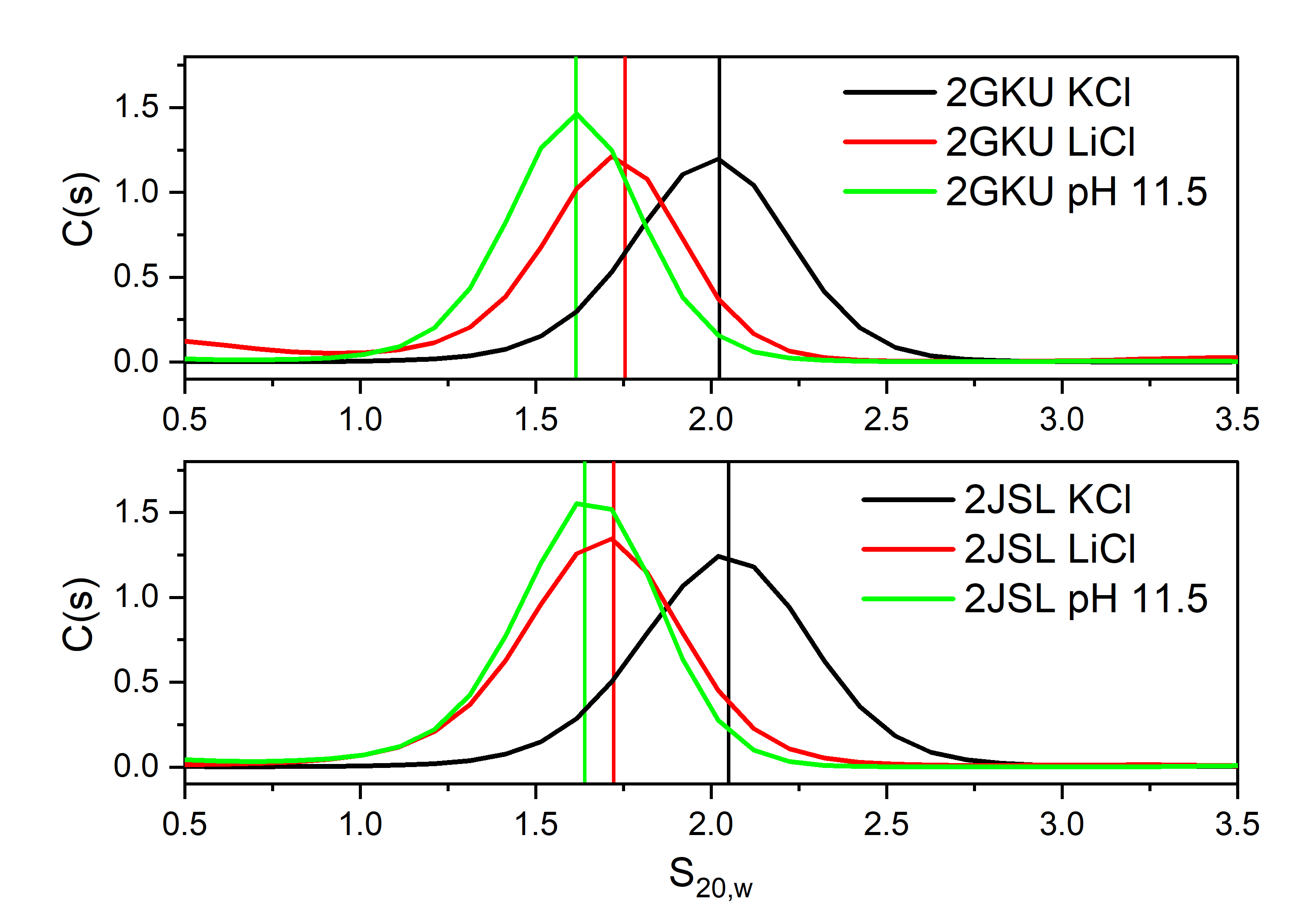


**Figure S3.** AUC-SV analysis of 2JSL under various buffer conditions showing a reduction in sedimentation coefficient in alkaline or LiCl conditions.


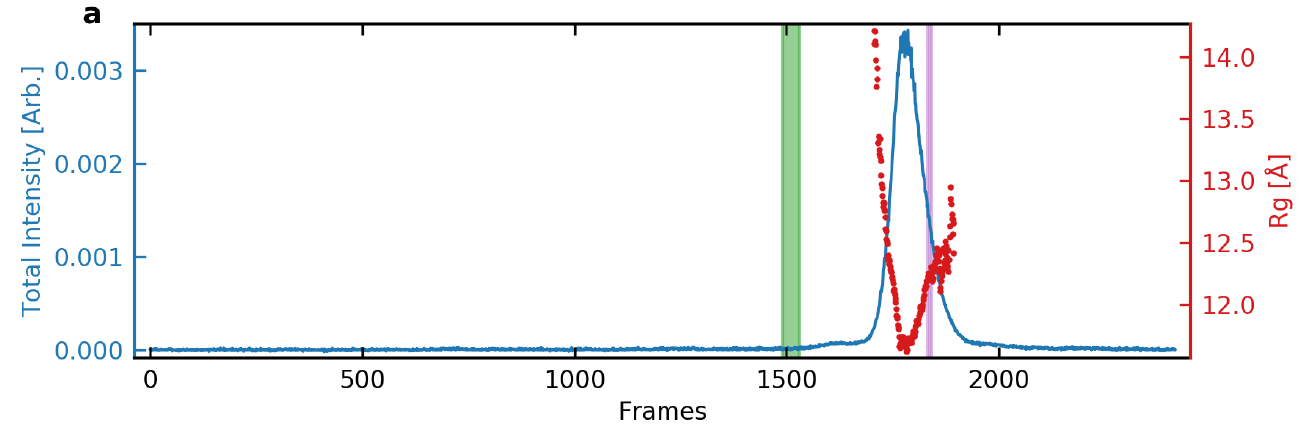


**Figure S4.** SEC-SAXS elution profile for 2GKU at pH 7.2 with KCl. Plot shows the series intensity (blue, left axis) vs. frame and R_g_ vs. frame (red, right axis). The green shaded region depicts the buffer region and purple shaded region shows the sample region used in subsequent analyses.


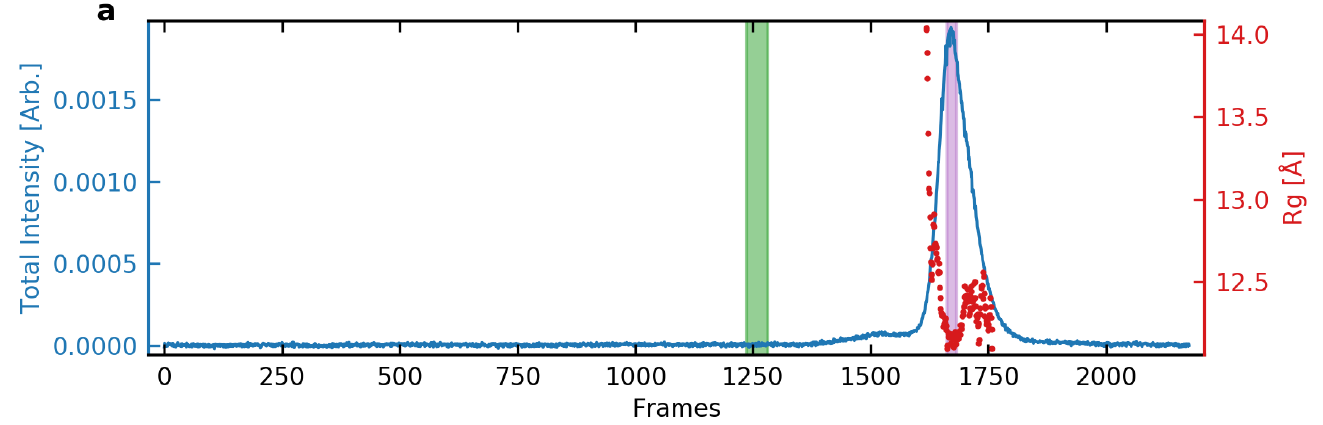


**Figure S5.** SEC-SAXS elution profile for 2JSL at pH 7.2 with KCl. Plot shows the series intensity (blue, left axis) vs. frame and R_g_ vs. frame (red, right axis). The green shaded region depicts the buffer region and purple shaded region shows the sample region used in subsequent analyses.


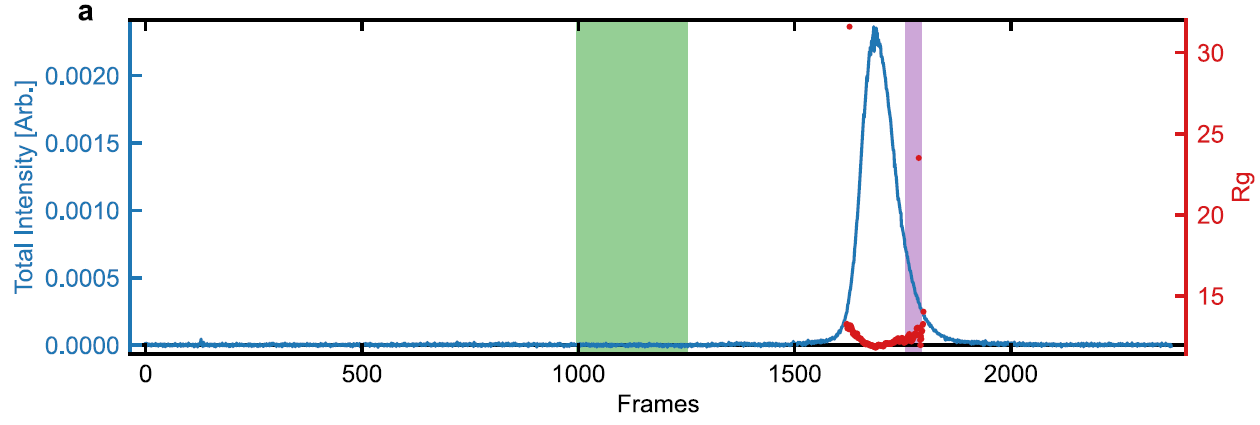


**Figure S6.** SEC-SAXS elution profile for 2GKU at pH 7.2 with LiCl. Plot shows the series intensity (blue, left axis) vs. frame and R_g_ vs. frame (red, right axis). The green shaded region depicts the buffer region and purple shaded region shows the sample region used in subsequent analyses.


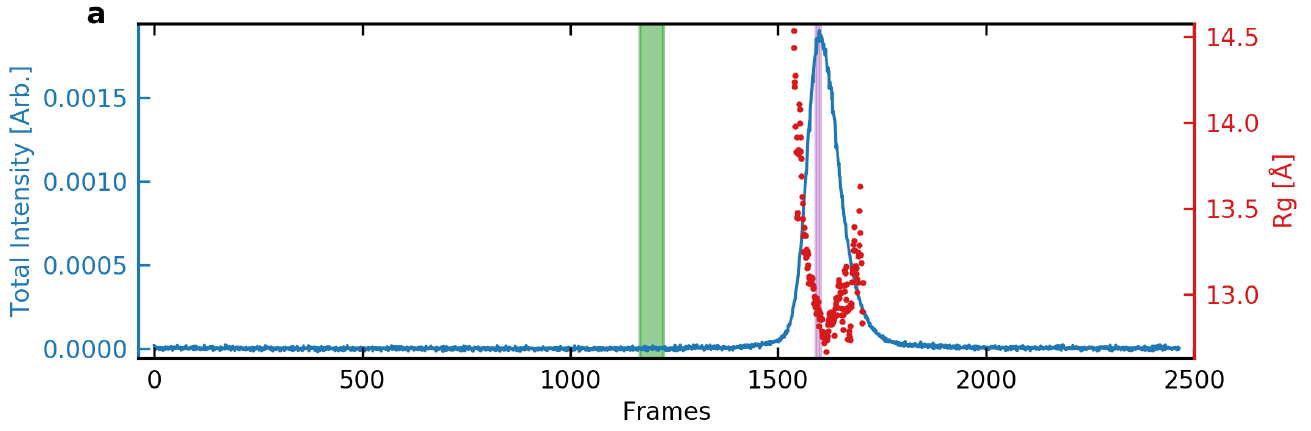


**Figure S7.** SEC-SAXS elution profile for 2JSL at pH 7.2 with LiCl. Plot shows the series intensity (blue, left axis) vs. frame and R_g_ vs. frame (red, right axis). The green shaded region depicts the buffer region and purple shaded region shows the sample region used in subsequent analyses.


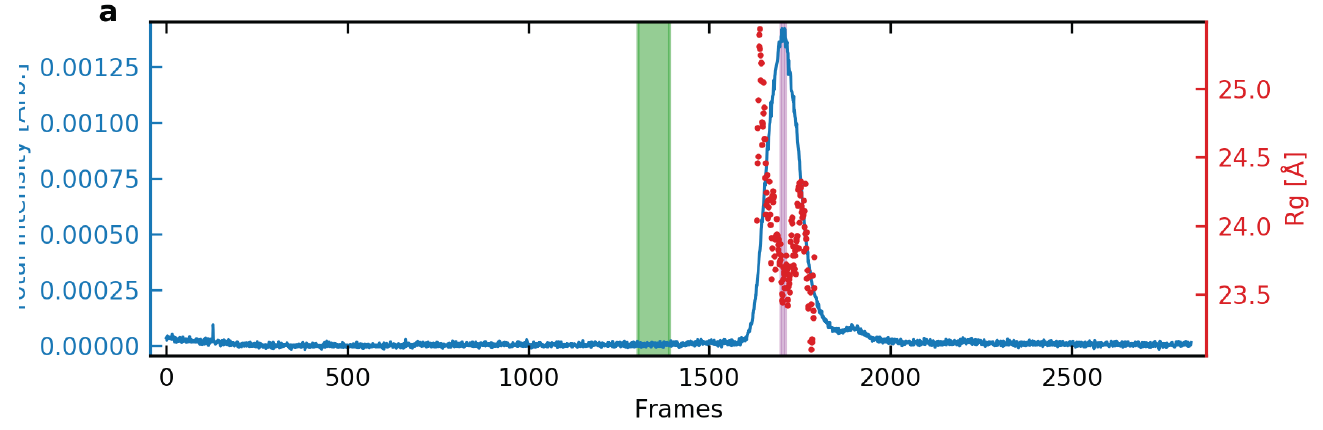


**Figure S8.** SEC-SAXS elution profile for 2GKU at pH 11.5 with KCl. Plot shows the series intensity (blue, left axis) vs. frame and R_g_ vs. frame (red, right axis). The green shaded region depicts the buffer region and purple shaded region shows the sample region used in subsequent analyses.


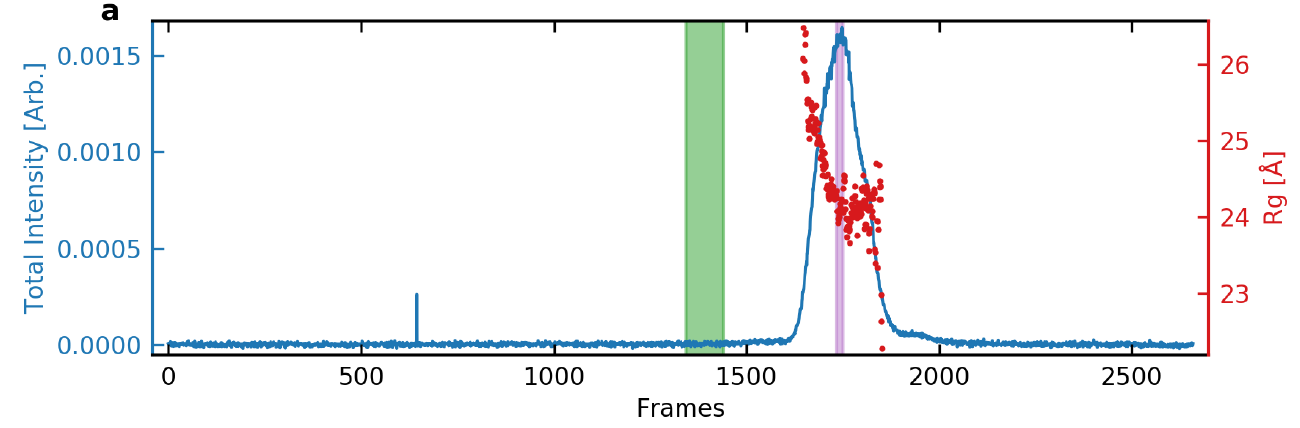


**Figure S9.** SEC-SAXS elution profile for 2JSL at pH 11.5 with KCl. Plot shows the series intensity (blue, left axis) vs. frame and R_g_ vs. frame (red, right axis). The green shaded region depicts the buffer region and purple shaded region shows the sample region used in subsequent analyses.

| (a) Sample Details. |  |  |  |  |  |  |
| --- | --- | --- | --- | --- | --- | --- |
|  | 2GKU pH 7.2 (KCl) | 2JSL pH 7.2 (KCl) | 2GKU pH 7.2 (LiCl) | 2JSL pH 7.2 (LiCl) | 2GKU pH 11.5 (KCl) | 2JSL pH 11.5 (KCl) |
| Organism | synthetic | synthetic | synthetic | synthetic | synthetic | synthetic |
| Source | IDT | IDT | IDT | IDT | IDT | IDT |
| Extinction coefficient (nearest neighbor approximation) (M^-1^ cm^-1^) | 244300 | 253100 | 244300 | 253100 | 244300 | 253100 |
| M from chemical composition (Da) | 7575 | 7879 | 7575 | 7879 | 7575 | 7879 |
| SEC-SAXS column, 10 x 300 Superdex 75 |  |  |  |  |  |  |
| Loading concentration (mg/mL) | 10.3 | 21.1 | 15 | 12.5 | 12.2 | 19.9 |
| Injection volume (μL) | 330 | 113 | 128 | 263 | 233 | 137 |
| Flow rate (mL/min) | 0.7 | 0.7 | 0.7 | 0.7 | 0.7 | 0.7 |
| Solvent (solvent blanks taken from SEC flow through prior to elution of protein) | 8 mM PO_4_^2-^, 185 mM KCl, 1 mM EDTA, pH 7.2 | 8 mM PO_4_^2-^, 185 mM KCl, 1 mM EDTA, pH 7.2 | 8 mM PO_4_^2-^, 185 mM LiCl, 1 mM EDTA, pH 7.2 | 8 mM PO_4_^2-^, 185 mM LiCl, 1 mM EDTA, pH 7.2 | 8 mM PO_4_^2-^, 185 mM KCl, 1 mM EDTA, pH 11.5 | 8 mM PO_4_^2-^, 185 mM KCl, 1 mM EDTA, pH 11.5 |
|  |  |  |  |  |  |  |
| (b) SAXS data-collection parameters. |  |  |  |  |  |  |
| Instrument/data processing | BioCAT facility at the Advanced Photon Source beamline 18ID with Pilatus3 1M (Dectris) detector | | | |  |  |
| Wavelength (Å) | 1.033 |  |  |  |  |  |
| Beam size (μm) | 150 (h) x 25 (ν) |  |  |  |  |  |
| Camera length (m) | 3.655 |  |  |  |  |  |
| q measurement range (Å^-1^) | 0.0044-0.35 |  |  |  |  |  |
| Absolute scaling method | N/A |  |  |  |  |  |
| Normalization | To incident intensity, by ion chamber counter | |  |  |  |  |
| Monitoring for radiation damage | Automated frame-by-frame comparison of relevant regions | |  |  |  |  |
| Exposure time, number of exposures | 0.5 s exposure time with a 2s total exposure period (0.5 s on, 1.5 s off) of entire SEC elution | | |  |  |  |
| Sample configuration | SEC-SAXS. Size separation by an AKTA Pure with a Superdex 75 Increase 10/300 GL column. SAXS data measured in a 1.5 mm ID quartz capillary | | | |  |  |
| Sample temperature (°C) | 22 |  |  |  |  |  |
|  |  |  |  |  |  |  |
| (c) Software employed for SAXS data reduction, analysis, and interpretation. |  |  |  |  |  |  |
| SAXS data reduction | Radial averaging; frame comparison, averaging, and subtraction done using BioXTAS RAW 2.1.1^[1]^ | | | |  |  |
| Extinction coefficient estimate | Nearest neighbor approximation | |  |  |  |  |
| Basic analyses: Guinier, P(r), V_p_ | Guinier fit, Kratky analysis, and molecular weight using BioXTAS RAW 2.1.1, P(r) function using PRIMUSqt (ATSAS v2.8.4^[2]^) | | | |  |  |
| Shape/bead modelling | N/A | | | |  |  |
| Atomic structure modelling | UCSF Chimera v1.11 & AMBER 2020 | | |  |  |  |
| Three-dimensional graphic model representations | UCSF Chimera v1.11 |  |  |  |  |  |
|  |  |  |  |  |  |  |
| (d) Structural parameters. |  |  |  |  |  |  |
| Guinier analysis | 2GKU pH 7.2 (KCl) | 2JSL pH 7.2 (KCl) | 2GKU pH 7.2 (LiCl) | 2JSL pH 7.2 (LiCl) | 2GKU pH 11.5 (KCl) | 2JSL pH 11.5 (KCl) |
| I(0) (cm^-1^) | 0.0112 ± 0.00002 | 0.0153 ± 0.00002 | 0.0166 ± 0.000008 | 0.0152 ± 0.00002 | 0.0178 ± 0.00005 | 0.0206 ± 0.00003 |
| R_g_ (Å) | 12.27 ± 0.04 | 12.13 ± 0.03 | 11.92 ± 0.01 | 12.89 ± 0.04 | 24.26 ± 0.17 | 24.26 ± 0.11 |
| qmin (Å^-1^) | 0.00586 | 0.00729 | 0.011 | 0.00872 | 0.00643 | 0.00843 |
| qR_g_ max | 1.2576 | 1.146 | 1.3099 | 1.2066 | 0.9949 | 1.0365 |
| Coefficient of correlation, R^2^ | 0.9796 | 0.9883 | 0.9988 | 0.9892 | 0.9776 | 0.9902 |
| SAXS MW (Ratio to Expected) (kDa) | 9.7 (1.28) | 9.9 (1.26) | 9.0 (1.19) | 9.5 (1.21) | 15.1 (1.99) | 15.7 (1.99) |
| P(r) analysis (GNOM) |  |  |  |  |  |  |
| I(0) (cm^-1^) | 0.0112 ± 0.00002 | 0.0154 ± 0.00002 | 0.00167 ± 0.0000103 | 0.0152 ± 0.00002 | 0.0178 ± 0.00004 | 0.0207 ± 0.00003 |
| Rg (Å) | 12.23 ± 0.03 | 12.20 ± 0.03 | 11.97 ± 0.02 | 13.18 ± 0.10 | 24.56 ± 0.11 | 25.03 ± 0.08 |
| D_max_ (Å) | 38 | 39 | 43 | 51 | 83 | 87 |
| ꭕ^2^ | 1.201 | 1.354 | 1.070 | 1.022 | 1.155 | 1.058 |
| Porod volume (Å^-3^) (ratio V_p_/calculated M) | 4500 | 4670 | 3920 | 4540 | 12400 | 12500 |
|  |  |  |  |  |  |  |
| (e) Shape model-fitting results |  |  |  |  |  |  |
|  | 2GKU pH 7.2 (KCl) | 2JSL pH 7.2 (KCl) | 2GKU pH 7.2 (LiCl) | 2JSL pH 7.2 (LiCl) | 2GKU pH 11.5 (KCl) | 2JSL pH 11.5 (KCl) |
| (f) Atomistic modelling. |  |  |  |  |  |  |
| Crystal structures/atomic coordinate files |  |  |  |  | Modeled | Modeled |
| q range for modelling |  |  |  |  | 0.00643-0.3 | 0.00843-0.3 |
| EOM GAJOE 2.1 (min ensembles = 5, max = 20, default parameters) |  |  |  |  |  |  |
| ꭕ^2^ |  |  |  |  | 1.162 | 1.126 |
| Rflex (random) / Rsigma |  |  |  |  | 84.25 (90.67) / 0.85 | 73.58 (87.66) / 0.63 |
| Constant subtracted |  |  |  |  | 0 | 0 |
| No. of representative structures |  |  |  |  | 9 | 3 |
| Final ensemble R_g_ (Å), D_max_ (Å) |  |  |  |  | 24.56, 76.94 | 25.54, 79.24 |
|  |  |  |  |  |  |  |
| (g) SASBDB IDs for data and models. |  |  |  |  |  |  |
| ID | SASDW43 | SASDW53 | SASDW63 | SASDW73 | SASDW83 | SASDW93 |

**Table S1.** Tabulated equilibrium SEC-SAXS data acquisition, reduction, analysis, results and SASBDB identifiers.

**
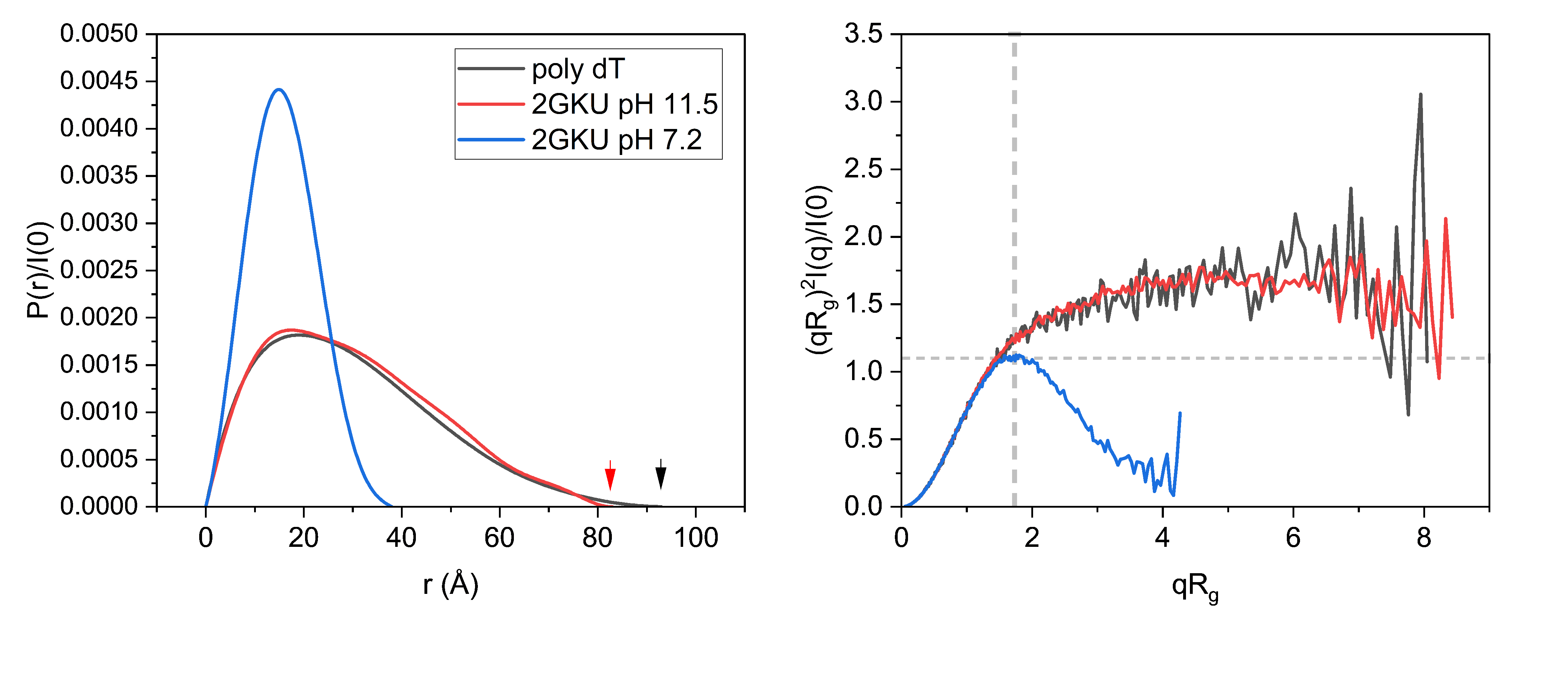
**

**Figure S10.** Normalized P(r) and Dimensionless Kratky plots comparing folded 2GKU in KCl at pH 7.2 (blue), pH 11.5 (red), and poly dT_24_ (black) at pH 7.2 with KCl.





**Figure S11.** (A) Scattering profiles, (B) Dimensionless Kratky plots, and (C) Normalized P(r) distributions of 2JSL in KCl at pH 7.2 (black), LiCl at pH 7.2 (red), and KCl at pH 11.5 (blue).


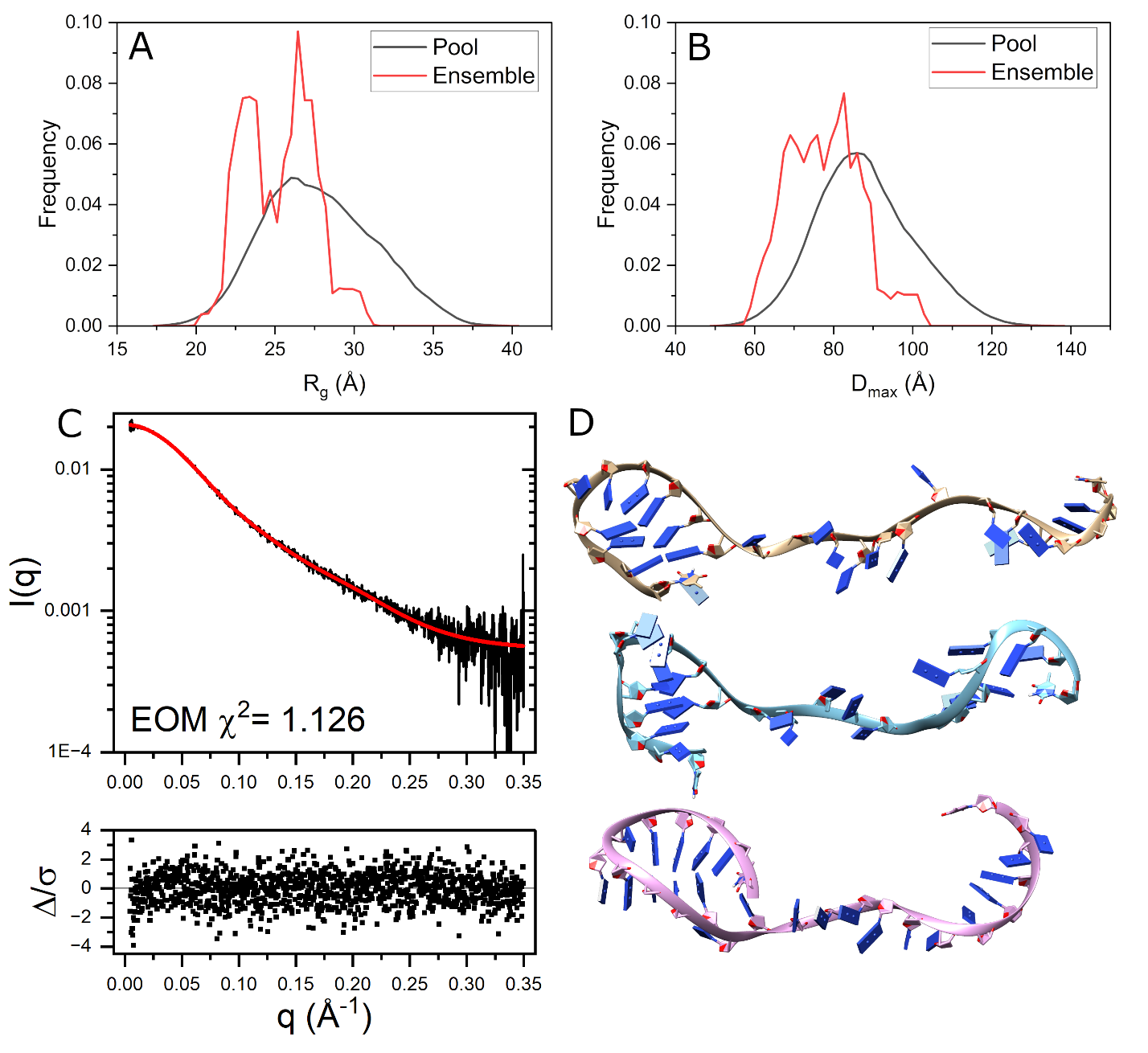


**Figure S12.** EOM analysis results for 2JSL. (A-B) EOM distributions for the radius of gyration (A) and D_max­_ (B) for the total pool of conformers (black) and the selected ensemble of flexible structures (red). (C) pH 11.5 scattering curve with EOM fit overlaid in red and residuals below. (D) Best fit ensemble of conformers chosen by EOM from duplicate 500 ns implicitly solvated MD simulations starting from single-stranded 2JSL showing the most extended (top) to the most compact conformations (bottom) oriented with 5’ end on the left. EOM statistics: R_flex_ (random) / R_sigma_: ~ 73.58% (~ 87.66%) / 0.63.


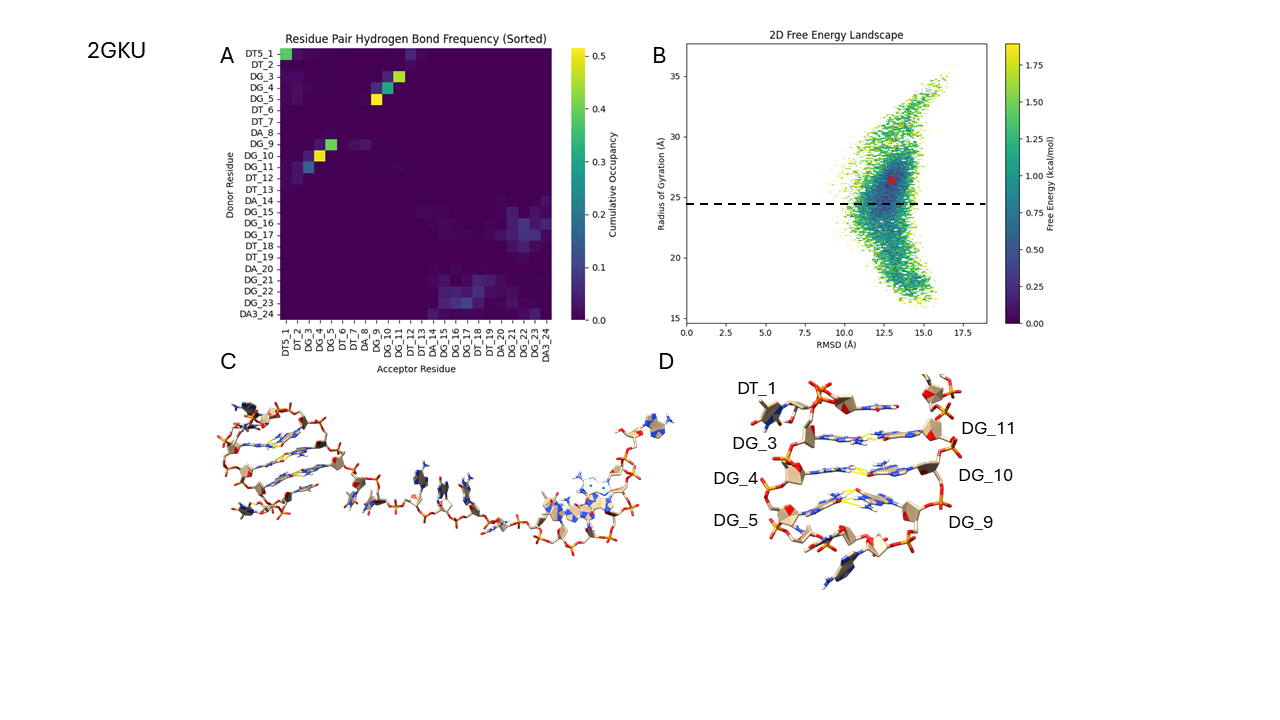


**Figure S13.** 2GKU MD trajectory analysis. (A) Residue pair hydrogen bond frequency analysis across the combined trajectories. Each residue pair interaction accounts for 1 or more h-bonds. (B) Pseudo 2D free energy landscape of the combined simulations showing the sampling distribution based on RMSD from first frame (x-axis) and radius of gyration (y-axis). The red star indicates the most frequently sampled or “global minimum” structure, and the dashed line corresponds to the measured radius of gyration for the alkaline denatured form from EQ-SAXS measurements. (C) and (D) show the full structure and close up of the 5’ guanine hairpin feature, respectively, of the model corresponding to the red star in (B). Yellow lines are indicative of H-bonds.


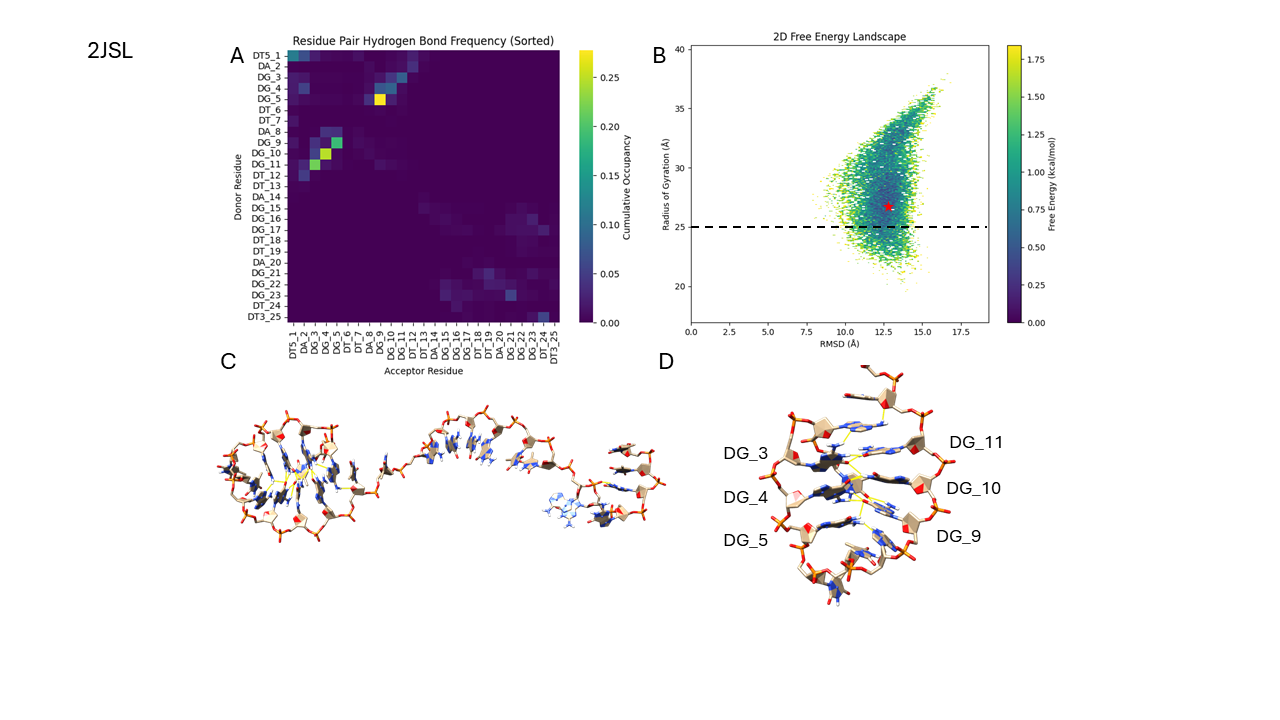


**Figure S14.** 2JSL MD trajectory analysis. (A) Residue pair hydrogen bond frequency analysis across the combined trajectories. Each residue pair interaction accounts for 1 or more h-bonds. (B) Pseudo 2D free energy landscape of the combined simulations showing the sampling distribution based on RMSD from first frame (x-axis) and radius of gyration (y-axis). The red star indicates the most frequently sampled or “global minimum” structure, and the dashed line corresponds to the measured radius of gyration for the alkaline denatured form from EQ-SAXS measurements. (C) and (D) show the full structure and close up of the 5’ guanine hairpin feature, respectively, of the model corresponding to the red star in (B). Yellow lines are indicative of H-bonds.

**
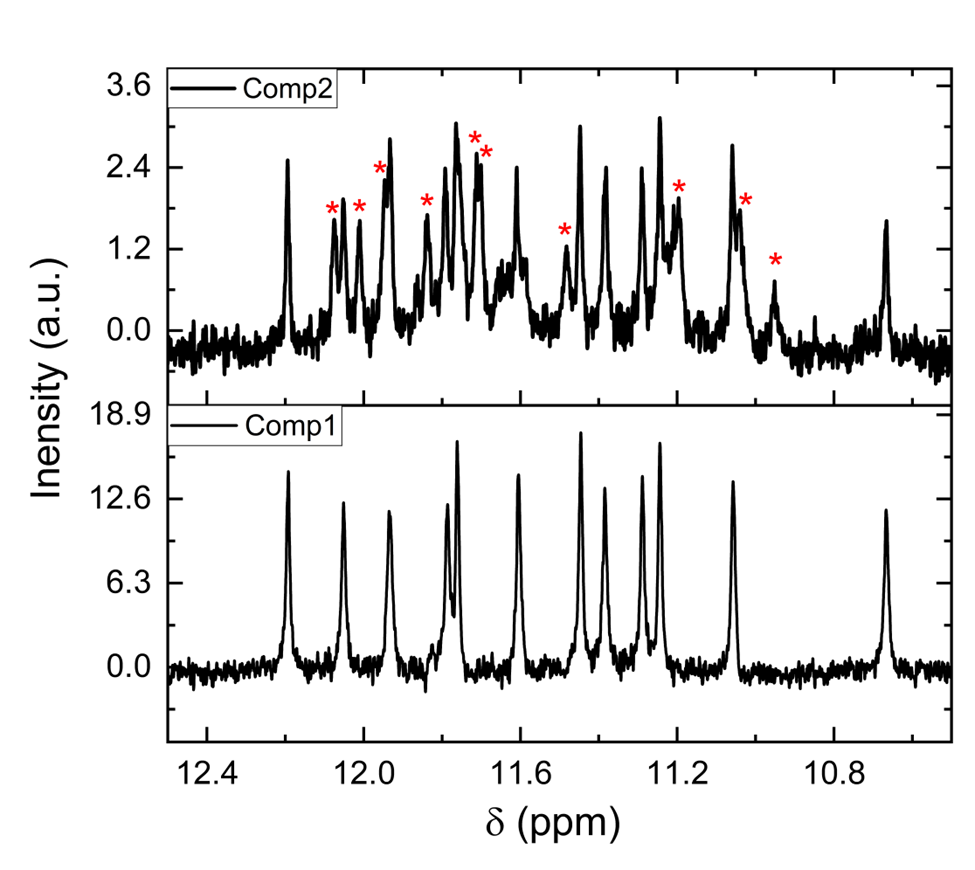
**

**Figure S15.** SVD results of hand-mixing pH jump 1D-NMR experiments showing the imino proton regions of the two major spectral components. Red asterisks indicate imino proton shifts that disappear rapidly after pH jump and may correspond to the early spectral intermediate observed by CD hand mixing in main text Figure 4. Component 1 is consistent with the imino shifts reported for the folded hybrid-1 2GKU.





**Figure S16.** Time course of R_g_ changes for 2GKU following an 11.5 to 7.2 pH jump. Experimental data (square symbols with error bars) are shown over the range of 0 – 1200 milliseconds. The solid line is the best least-squares fit to an exponential decay with a time constant of 187 ± 13 milliseconds. The top panel shows the residuals of the fit. Red and blue dashed lines indicate the EQ-SAXS measured R_g_ values for the alkaline denatured and pH 7.2 equilibrated 2GKU, respectively.





**Figure S17.** Time-resolved SAXS results of 2JSL following pH jump from 11.5 to 7.2. (A) Selected normalized P(r) distributions of the pH-induced structural collapse of 2GKU bracketed by the equilibrium SAXS profiles showing the conversion from an extended unstructured species to a globular and compact particle of nearly identical size and shape as the folded hybrid 1 form. (B) Dimensionless Kratky plots (data re-binned for clarity with log mode and re-bin factor 4) showing the transition from the denatured flexible chain to a compact globular form that is nearly identical to the equilibrium 2GKU scattering. (C) REGALS derived regularized P(r) distributions comparing the two deconvoluted components. (D) Regularized component concentration profiles from REGALS deconvolution with single exponential decay relaxation times overlaid and fit shown in blue. Red and black curves correspond to the solid red and black P(r) distributions in C.

**REFERENCES**

[1] J. B. Hopkins, *bioRxiv* **2023**.

[2] D. Franke, M. V. Petoukhov, P. V. Konarev, A. Panjkovich, A. Tuukkanen, H. D. T. Mertens, A. G. Kikhney, N. R. Hajizadeh, J. M. Franklin, C. M. Jeffries, D. I. Svergun, *J Appl Crystallogr* **2017**, *50*, 1212-1225.
